# Effect of Bi-functional Hierarchical Flower-like CoS Nanostructure on its Interfacial Charge Transport Kinetics, Magnetic and Electrochemical Behaviors for Supercapacitor and DSSC Applications

**DOI:** 10.1038/s41598-018-37463-0

**Published:** 2019-02-04

**Authors:** K Ashok Kumar, A Pandurangan, S Arumugam, M Sathiskumar

**Affiliations:** 1 Department of Chemistry, Anna University, Chennai, 600025 Tamil Nadu India; 2 Centre for High Pressure Research, School of Physics, Bharathidasan University, Tiruchirappalli, 620024 Tamil Nadu India

## Abstract

Metal sulfides are of great interest for future electrode materials in supercapacitor and solar cell applications owing to their superior electrochemical activity and excellent electrical conductivity. With this scope, a binary transition metal sulfide (CoS) is prepared via one-step hydrothermal synthesis. Hexagonal phase of CoS with space group of P6_3_/mmc(194) is confirmed by XRD analysis. Additional cubic Co_3_S_4_ phase in the prepared sample originates the mixed valence state of Co (Co^2+^ and Co^3+^) is affirmed from XPS analysis. Morphological features are visualized using HRSEM images that shows nanoflower shaped star-anise structure. Employing the prepared CoS as active electrode material, interfacial charge transport kinetics is examined by EIS-Nyquist plot. The supercapacitive performances are tested in two and three-electrode system which exhibited respective specific capacitances of 57 F/g and 348 F/g for 1 A/g. Further, the fabricated asymmetric CoS//AC supercapacitor device delivers an appreciable energy density of 15.58 Wh/kg and power density of 700.12 W/kg with excellent cyclic stability of 97.9% and Coulombic efficiency of 95% over 2000 charge-discharge cycles. In addition, dye-sensitized solar cells are fabricated with CoS counter electrode and the obtained power conversion efficiency of 5.7% is comparable with standard platinum based counter electrode (6.45%). Curie-Weiss plot confirms the transition of paramagnetic nature into ferrimagnetic behavior at 85 K and Pauli-paramagnetic nature at 20 K respectively. Temperature dependent resistivity plot affirms the metallic nature of CoS sample till 20 K and transition to semiconducting nature occurs at <20 K owing to Peierl’s transition effect.

## Introduction

Transition metal sulfides have been widely investigated as active electrode materials for energy-related applications including fuel cells, lithium-ion batteries, photovoltaic devices and electrochemical capacitors owing to their rich in physico-chemical properties^1–3^. Especially, cobalt sulfide has significant interest due to its abundance in nature, low cost, good electrical conductivity and high electrocatalytic activity. Cobalt sulfide with variety of stoichiometries including CoS, CoS_2_, Co_3_S_4_ and Co_9_S_8_ has been extensively used in supercapacitor application owing to their excellent electrochemical stability, high redox activity, superior capacitive properties and relatively good cyclic stability^4–8^. In addition, cobalt sulfide has been proved to be very effective in catalyzing the redox electrolyte in dye-sensitized solar cells (DSSCs) and exhibiting great potential to replace the traditional noble metal platinum (Pt) counter electrodes in DSSCs. This excellent electrochemical performance and catalytic behaviour makes CoS as a promising electrode material in supercapacitors and DSSC applications. As for application in DSSC, Srinivasa Rao *et al*. prepared porous cobalt sulfide by electrodeposition method and reported a maximum power conversion efficiency of 5.32%^9^. Using CoS_2_ as counter electrode material, the photoconversion efficiency of 4.16% was attained by Matthew *et al*.^10^.

Up to date, many reports are available on the usage of cobalt sulfide electrode for supercapacitor application (summarized in Table S2 of Supplementary Information). Bo You *et al*. synthesized hollow CoS nanoprisms by microwave and solvothermal method with respective specific capacitances of 224 F/g and 156 F/g at current density of 1 A/g^4^. Jia-Chao Xing showed a specific capacitance of 237 F/g at current density of 1 A/g for hydrothermal synthesis of octahedran shaped CoS_2_^11^. Using two step hydrothermal route Houzhao *et al*. reported the preparation of Co_9_S_8_ hollow nanotubes which exhibited a specific capacitance of 245 F/g at 1 A/g^12^. Hongyu Chen *et al*. synthesized 3D flower-like CoS hierarchitectures by recycling method and reported a specific capacitance of 409.3 F/g at 1 A/g^13^. Han Hu *et al*. prepared the nanoparticle assembled nanoboxes surrounded by outer CoS nanosheets via metal organic framework approach which presented a high specific capacitance of 980 F/g in three electrode system and 118 F/g in two electrode device respectively for 1 A/g current density^14^. Ye Li *et al*. reported the hydrothermal synthesis of flower like Co_1-x_S which presented a specific capacitance of 674 F/g at current density of 3 A/g^15^. Hollow structured Co_1-x_S was synthesized by C. Ranaveera *et al*. through hydrothermal method and showed a specific capacitance of 420 F/g at 1 A/g^16^. Sphere like CoS prepared by P.Justin *et al*. via hydrothermal method showed a specific capacitance of 349 F/g at 1 A/g^8^. Fulian Luo *et al*. reported a specific capacitance of 586 F/g at 1 A/g using hierarchical flower shaped CoS prepared by microwave assisted heating method^17^.

Using two-step hydrothermal route, R.B. Rakhi *et al*. prepared the asymmetric supercapacitor devices based on Co_9_S_8_ nanoflakes and Co_9_S_8_ octahedral which resulted a specific capacitance of 83 F/g and 18.6 F/g at 1.25 A/g and 1 A/g respectively^7^. Also, synthesis of hierarchical porous nanocoral like Co_3_S_4_ by hydrothermal method was reported by Guijing Liu *et al*. The prepared porous Co_3_S_4_ based supercapacitor device possesses a specific capacitance of 132.7 F/g at 1 A/g in 2-electrode device configuration^6^. Recently, Subramani *et al*., reported the preparation of dumb-bell shaped CoS for the application of supercapacitance and realized the specific capacitance of 310 F/g (5 A/g) and 47 F/g (2 A/g) in 3 and 2 electrode configuration respectively^18^. Although the previous research results reported that the cobalt sulfide with high energy density has been used as an excellent electrode material for supercapacitors, the power density and long term cyclic stability is still need to be further improved.

On the other hand, the morphology of electrode material also plays crucial role in determining the electrochemical behaviour and interfacial charge transport kinetics. So far, various methods have been devoted to synthesis CoS nanostructures with different morphologies including nanowires^19^, hollow spheres^20^, ellipsoids^21^, nanosheets^22^ and micro-flowers^17,23,24^. In particular, recent efforts have been invested towards the preparation and characterization of complex micro/nano structures, especially three-dimensional (3D) hierarchical architecture assembled by low dimensional building blocks such as nanoparticles, nanorods, nanowires and nanosheets^25–27^. Because, such hierarchical morphologies can provides high surface area for more electrolyte diffusion which is beneficial for high redox activity, high electron transfer and reduced recombination dynamics. Generally, the synthetic methods to produce hierarchical micro/nano structures necessitate complex procedures such as usage of toxic or expensive chemicals, high temperature, structure directing templates and time consuming growth process. Hence, a challenge still persists to develop a simple and effective technique for the fabrication of hierarchical CoS architectures with different building blocks.

**Figure 1:**
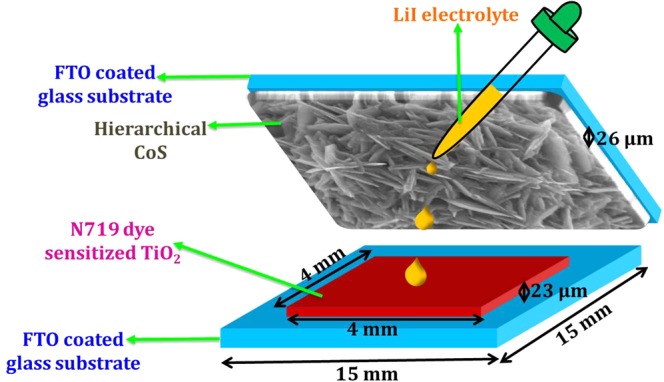
Schematic representation of fabricated DSSC based on hierarchical CoS counter electrode.

To pursuit this challenge, we report a facile one step hydrothermal synthesis of 3D hierarchical flower like CoS nanostructure and investigate the prepared material for supercapacitor and DSSC applications. The electrochemical properties such as interfacial charge transport kinetics, galvanostatic charge-discharge profile and cyclic voltammetric analysis in 2 and 3 electrode system were investigated in detail. The prepared CoS film was utilized as counter electrode in DSSC application (scheme of constructed DSSC in Fig. 1) and realized the power conversion efficiency of 5.7%. The constructed asymmetric supercapacitor device based on hierarchical CoS exhibited excellent electrochemical properties with remarkably high power density and cyclic stability. In addition, temperature dependent magnetic transition and transport behaviour of the prepared CoS are also explored in this paper.

## Results and Discussions

### Structural Analysis: X-Ray Diffraction

**Figure 2:**
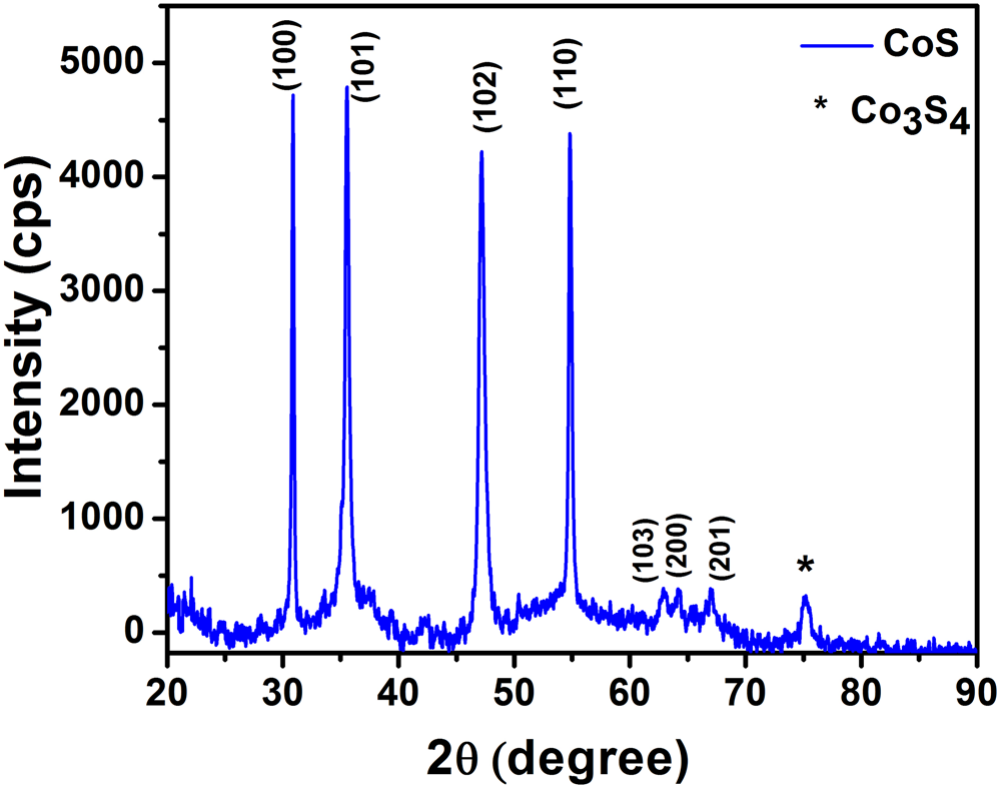
XRD pattern of prepared CoS.

X-Ray diffraction pattern confirms the formation of hexagonal phase of CoS with the space group of P6_3_/mmc(194). Diffraction peaks appeared at 30.8^°^, 35.5^°^, 47.2^°^, 54.8^°^, 62.9^°^, 64.1^°^ and 66.9^°^ corresponds to (100), (101), (102), (110), (103), (200) and (201) planes of hexagonal CoS (Fig. 2) are well matched with the standard JCPDS data: 65-3418^18^. The obtained peaks were fitted with XRDA 3.1 software and the estimated lattice parameters were found to be a = b = 3.3463 ± 0.0003 Å (3.368 Å) and c = 5.1476 ± 0.0004 Å (5.170 Å) with the cell volume of  V= 49.986 Å^3^ (50.79 Å^3^). The average crystallite size of the prepared CoS is calculated to be 46 nm using Scherrer’s equation as follows:

1 $${\rm{D}}=k{\rm{\lambda }}/{\rm{\beta }}\,\mathrm{Cos}\,{\rm{\theta }}$$

where, D is average crystallite size, k is shape factor, β is full width at half maximum of each diffraction peak and θ represents the diffraction angle. In addition to hexagonal CoS phase, the peak appeared at 75.1^°^ vouch for the existence of additional cubic phase (JCPDS No: 73–1703) of cobalt sulfide (Co_3_S_4_). Presence of cubic Co_3_S_4_ phase instigate the lattice strain and a defect in the prepared CoS sample^28^. To measure the induced lattice strain, Williamson-Hall plot (Fig. S2 of Supplementary Information) was derived, where 4sinθ is plotted against βcosθ. The estimated lattice strain is found to be 0.00439 and the calculated crystallite size after the correction of strain induced broadening is 67 nm (from W-H plot).

### Morphological Analysis: HRSEM

**Figure 3:**
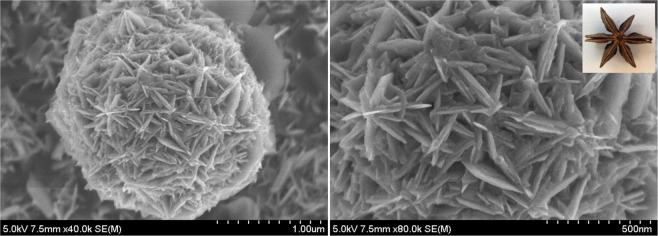
HRSEM images of CoS.

The surface morphology of prepared CoS exhibits hierarchical nanostructured flower like morphology shown in Fig. 3. It can be observed that the prepared CoS hierarchical flower is comprised of large number of nanosheet like petals with thickness around 40–50 nm. The formation of hierarchical CoS resembles with structure of star anise flower (Inset of Fig. 3) with meso/macropores in between the nanosheet like petals. This type of three dimensional hierarchical porous structure can provide high surface area which resulted to have effective contact between the CoS electrode and electrolyte ions. Also, the porous structure of hierarchical CoS flower can offer more number of interfacial and electrocatalytic active sites leading to the enhancement of electrochemical behaviors^29^. Hence, the prepared hierarchical CoS flower can be useful for active electrode material in supercapacitors and DSSC applications.

### Formation Mechanism

**Figure 4:**
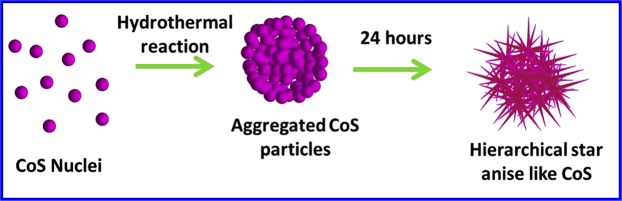
Formation mechanism of hierarchical nanostructured CoS.

To understand the possible formation of hierarchical flower like cobalt sulfide, the growth mechanism was proposed and the schematic representation was illustrated in Fig. 4. At the initial stage of hydrothermal reaction, the Co^2+^ and S^2−^ ions released by hydrolysis of CoCl_2_.6H_2_O and CH_4_N_2_S reacted to form the CoS nuclei. With the proceeding of reaction, the tiny nuclei aggregated into larger particles. When the reaction time was further prolonged, the aggregated particles are grown into hierarchical star anise like CoS flowers by Ostwald ripening process.

$${{\rm{CoCl}}}_{{\rm{2}}}.{{\rm{6H}}}_{{\rm{2}}}{\rm{O}}+{{\rm{2H}}}_{{\rm{2}}}{\rm{O}}\to {\rm{Co}}{({\rm{OH}})}_{{\rm{2}}}+{{\rm{6H}}}_{{\rm{2}}}{\rm{O}}+{\rm{2HCl}}$$

$${\rm{Co}}{({\rm{OH}})}_{{\rm{2}}}+{{\rm{H}}}_{{\rm{2}}}{\rm{O}}\to {{\rm{Co}}}^{2+}+{{\rm{H}}}_{{\rm{2}}}{\rm{O}}+{{\rm{2OH}}}^{-}$$

$${{\rm{CH}}}_{{\rm{4}}}{{\rm{N}}}_{{\rm{2}}}{\rm{S}}+{{\rm{2H}}}_{{\rm{2}}}{\rm{O}}\to {{\rm{2NH}}}_{{\rm{3}}}+{{\rm{H}}}_{{\rm{2}}}{\rm{S}}+{{\rm{CO}}}_{{\rm{2}}}$$

$${{\rm{H}}}_{{\rm{2}}}{\rm{S}}+{{\rm{2H}}}_{{\rm{2}}}{\rm{O}}\to {{\rm{2H}}}_{{\rm{3}}}{{\rm{O}}}^{+}+{{\rm{S}}}^{{\rm{2}}-}$$

$${{\rm{C}}{\rm{o}}}^{2+}+{{\rm{S}}}^{2-}\to {\rm{C}}{\rm{o}}{\rm{S}}$$

### Elemental Analysis: X-Ray Photoelectron spectroscopy

**Figure 5:**
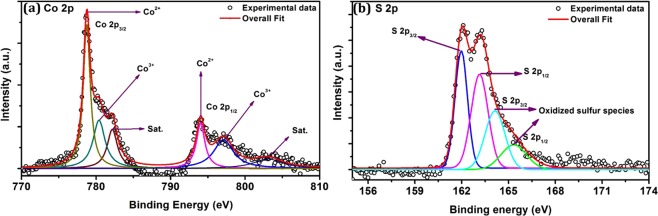
X-Ray photoelectron spectrum. (**a**) Deconvoluted core level spectra of Co-2p and (**b**) S-2p.

The valence state of elements and the surface chemical composition of the prepared CoS was evaluated using X-ray photoelectron spectroscopy. Figure 5 shows the high resolution XPS core level spectra of Co-2p and S-2p. The spin-orbital splitting of Co-2p peaks are deconvoluted into two main peaks, two low intense peaks with associated satellite peaks^30^ (Fig. 5a). The two main peaks at 778.77 eV and 794.06 eV correspond to Co-2p_3/2_ and Co 2p_1/2_. The binding energy separation between Co-2p_1/2_ and Co-2p_3/2_ was found to be 15.29 eV, which is in good agreement with reported values^30^. The main and shake up peaks appeared at high binding energies of 778.77 eV and 782.15 eV respectively is attributed to the presence of Co^2+^ which affirmed with reported values^11,17^. The additional two low intense peaks appeared at 780.38 eV and 797.02 eV with the spin-orbit separation of 16.64 eV are attributed to Co^3+^ valence state owing to the existence of additional cubic Co_3_S_4_ phase. Generally, the hexagonal CoS can formed with single Co^2+^ valance state. But the presence of additional cubic Co_3_S_4_ phase in the prepared sample can introduce charge imbalance and induce a defect in the hierarchical CoS. Hence, to compensate the charge balance some of Co^2+^ was converted into Co^3+^ valence state^24^. Figure 5b shows the deconvoluted core level spectrum of S-2p which exhibits four peaks. The peak observed at 161.97 eV corresponds to S 2p_3/2_ which is associated with the metal-sulfide (Co-S) bonding. The S 2p_1/2_ peak appeared at 163.15 eV is attributed to surface bonding of divalent sulfur (S^2−^)^31^. In addition, the peak positioned at high binding energy of 164.12 eV (S 2p_3/2_) and 165.29 eV (S 2p_1/2_) are consistent with the oxidation of sulfur content (elemental sulfur) in the prepared CoS^6,32^.

### Optical Studies: UV-DRS

**Figure 6:**
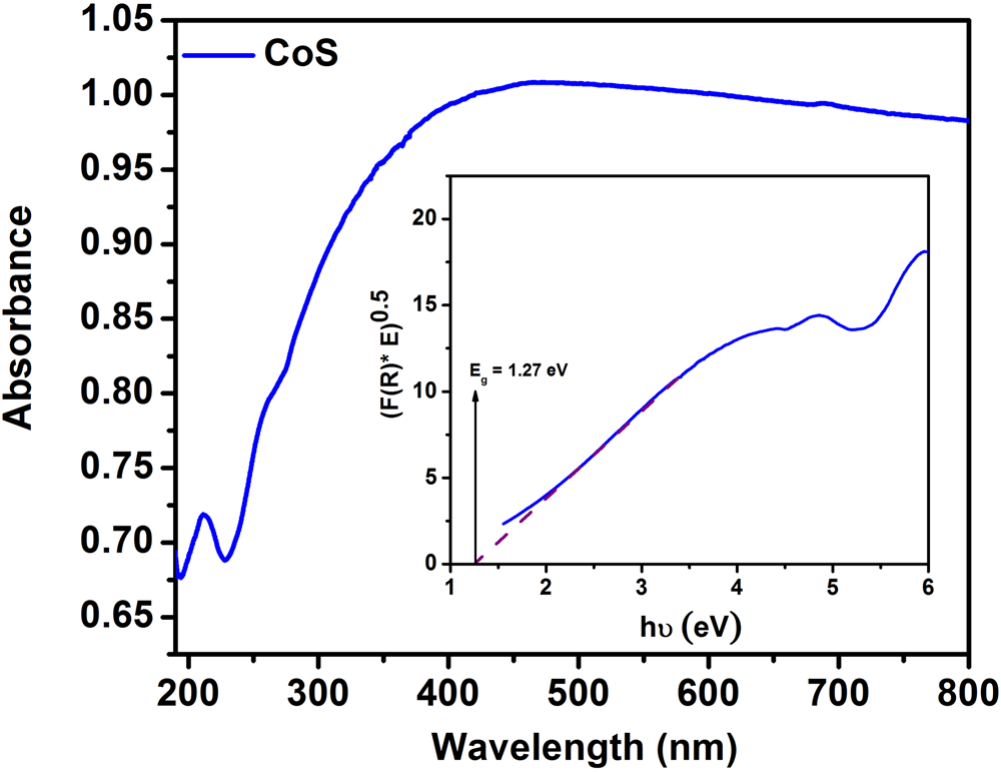
UV-DRS spectrum of CoS (Inset shows Kubelka-Munk function).

The optical property of prepared CoS was studied using UV-DRS analysis. Figure 6 shows the absorption spectrum of prepared CoS which reveals a wide range of absorption in the visible region. Using Kubelka-Munk function, the estimated bandgap energy is found to be 1.27 eV (Inset of Fig. 6). In addition, the optical absorption of prepared CoS are extended to near-infrared (NIR) regime which can enhance the current density of fabricated DSSC based on CoS counter electrode. Improved absorption in visible and NIR region can produce enhanced conduction process under light illumination which is evidenced with the photoconductivity studies (given as Fig. S8 of Supplementary Information).

### Electrochemical Analysis: Cyclic Voltammetry

#### Positive electrode materials

**Figure 7:**
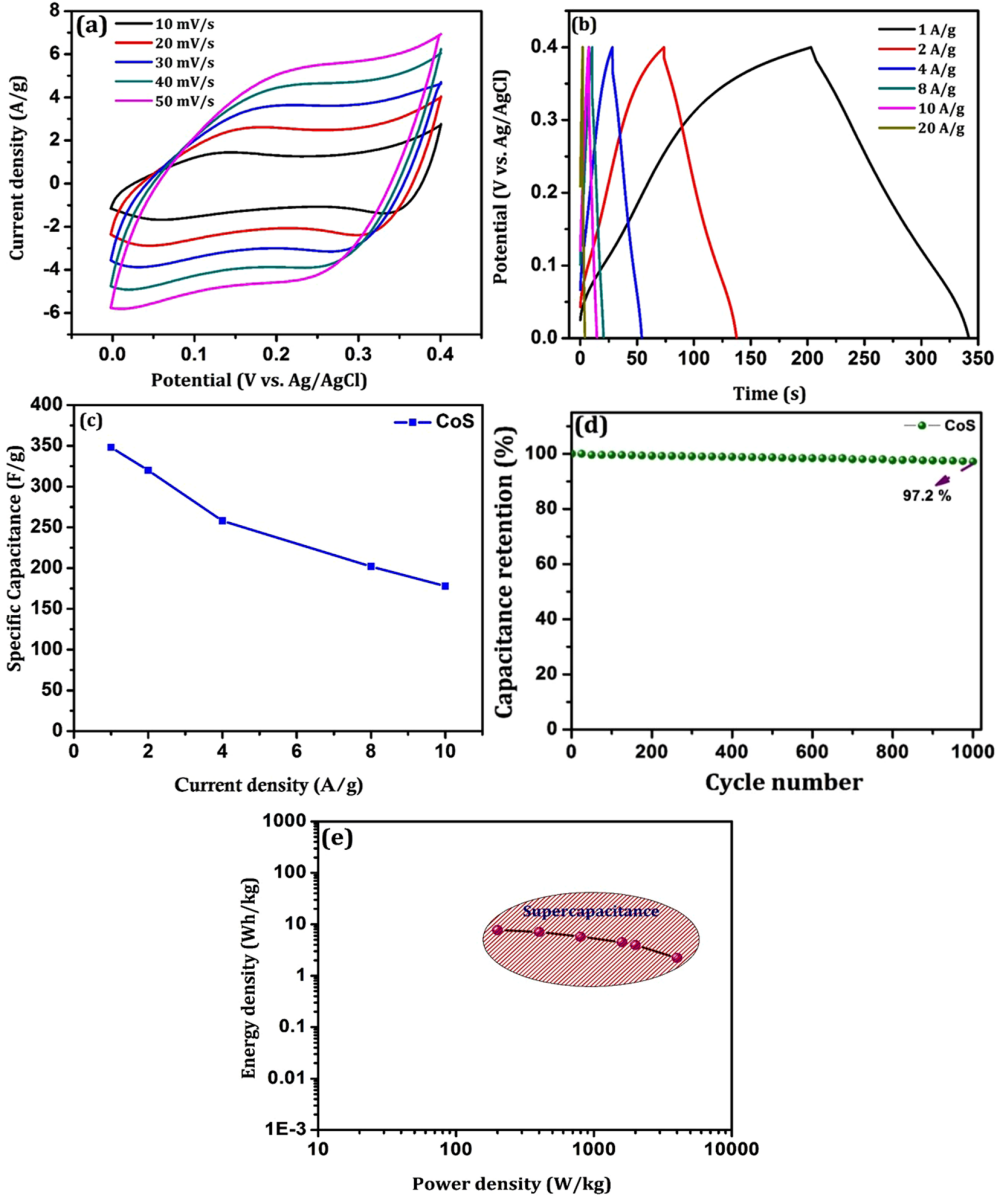
Electrochemical measurements in three electrode system. (**a**) Cyclic voltammetry at different scan rates, (**b**) Galvanostatic charge-discharge profile, (**c**) specific capacitance (**d**) cyclic stability and (**e**) Energy-Power density relation (Ragone plot) of CoS.

The electrochemical properties of the prepared CoS were examined using cyclic voltammetry (CV) from the potential range of 0 to 0.4 V (vs. Ag/AgCl) in three electrode system with 6 M KOH as supporting electrolyte. Figure 7a shows the CV curves of CoS electrode at scan rate of 10 to 50 mV/s which clearly show one distinct pair of redox peaks during the anodic and cathodic sweeps. It indicates the pseudo-capacitive characteristics of the prepared material attributing to reversible Faradic reactions within the electrode material interior. Since sulfur is in the same family as oxygen, the redox reaction of CoS in KOH electrolyte is similar to the redox reaction mechanism of CoO which is termed in the following equation,

$$\mathrm{CoS}\,+{{\rm{OH}}}^{-}\leftrightarrow {\rm{CoSOH}}+{{\rm{e}}}^{-}$$

$${\rm{CoSOH}}+{{\rm{OH}}}^{-}\leftrightarrow {\rm{CoSO}}+{{\rm{H}}}_{{\rm{2}}}{\rm{O}}+{{\rm{e}}}^{-}$$

Here, the anodic peaks are attributed to oxidation of CoS to CoSOH and cathodic peaks are due to reverse redox process at the CoS electrode. In addition, at low scan rates the anodic and cathodic peaks are identical and explicit, which reveals that the prepared CoS exhibits good rate capability. Further, the electrochemical capacitive behavior occurred here is contributed by the reversible electron transport process of the mixed valence redox couple (Co^2+^/Co^3+^) in alkaline redox mediator^33^.

#### Charge-discharge curve and cyclic stability

To evaluate the specific capacitance (C_s_), the prepared hierarchical CoS coated electrode was galvanostatically charged and discharged at the window potential of 0 to 0.4 V vs. Ag/AgCl in alkaline electrolyte (6 M KOH). Figure 7b shows the charge-discharge profile at various current densities which is in accordance with the CV results. The specific capacitance is calculated using the relation,

2 $${{\rm{C}}}_{s}={\rm{I}}\,{\rm{x}}\,{\rm{\Delta }}t\,/\,({\rm{m}}\,{\rm{x}}\,{\rm{\Delta }}{\rm{V}})$$

where, C_s_ represents the specific capacitance (F/g), I is the discharge current density (A), Δt is the discharge time (s), ΔV is the window potential (V) and m is the mass of active electrode material (2.2 mg). The specific capacitance calculated from each discharge curve is found to be 348 F/g, 242 F/g, 178 F/g and 100 F/g at corresponding current densities of 1 A/g, 5 A/g, 10 A/g and 20 A/g. Figure 7c represents the specific capacitance of prepared CoS electrode as a function of current densities. In the present study, the obtained 178 F/g for 10 A/g current density is nearly 51% of its initial capacitance of 348 F/g at 1 A/g which may accepted as reasonable capacitance value even at high current density. The attained specific capacitance for prepared CoS is due to the enhanced interfacial contact by the hierarchical structure of CoS with open pores. The maximum specific capacitance of 348 F/g obtained at the current density of 1 A/g is comparable with the previous reported results (Table S2 of Supplementary Information). Furthermore, to examine the cyclic stability of the CoS electrode, the charge-discharge measurement was carried out at a constant current density of 3 A/g over 1000 cycles. The resultant specific capacitance after 1000 cycles of charge-discharge profile retains 97.2% of its initial C_s,_ which delivers high cyclic stability of prepared hierarchical CoS (Fig. 7d). A typical Ragone plot provides energy and power density relation shown in Fig. 7e. From the plot, it was confirmed that the obtained energy density (7.73 Wh/kg) and power density (200 W/kg) of the prepared CoS electrode for 1 A/g current density was comparable with the existing supercapacitors (Table S2 of Supplementary Information).

#### Electron transport Properties: EIS-Nyquist plot

**Figure 8:**
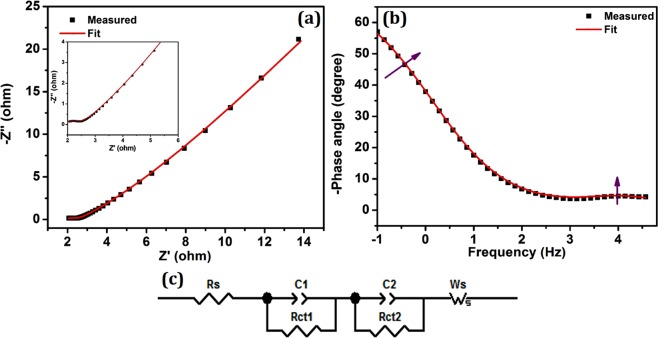
Electrochemical impedance spectroscopy. (**a**) Nyquist plot of CoS in three electrode system (Inset shows enlarged view of high frequency semicircle), (**b**) Bode-phase plot and (**c**) Equivalent circuit model for fitting.

Nyquist plot of electrochemical impedance spectroscopy (EIS) was carried out in three electrode configuration to examine the interfacial charge transport dynamics of prepared CoS electrode. Analysis was done using electrochemical workstation for an applied open circuit potential (OCP) with frequency range from 1 MHz to 0.1 Hz at room temperature. For the electrochemical measurement, hierarchical CoS coated carbon felt was employed as working electrode, Ag/AgCl as reference electrode and platinum wire (Pt) as counter electrode were placed in 6 M of KOH as supporting electrolyte. Figure 8a shows the Nyquist plot of EIS spectrum which reveals double semicircle behavior with the maximum phase shift at high and mid frequency regions. Inset of Fig. 8a clearly shows the enlarged view of high frequency semicircle. In addition, the existence of two phase shifts in Bode phase plot (Fig. 8b) further confirms the presence of two interfacial charge transport resistances^34^. The obtained semicircles were fitted with an equivalent circuit modelling (Fig. 8c) and the fit parameters are tabulated in Table 1. Semicircle at high frequency region gives charge transport resistance (R_ct1_) at CoS/electrolyte interface and semicircle at mid frequency region provides charge transport resistance (R_ct2_) at grain interior of CoS electrode. In addition, R_s_ corresponds to surface sheet resistance of carbon felt electrode used in the analysis, C_μ_ is the total chemical capacitance of electrode material while applying the potential and W_s_ represents the Warburg diffusion resistance of electrolyte which is found to be very low.

**Table 1:** Electrochemical parameters from EIS-Nyquist plot in three electrode system.

Sample	R_s_(Ω)	R_ct1_(Ω)	R_ct2_(Ω)	W_s_ (Ω)	τ(s)	χ^2^
CoS	1.87	0.60	50.43	0.0976	0.11	6.991E-5

From Table 1, it can be noted that R_ct1_ is very low compared with R_ct2_ which implies the efficient electron transportation between electrolyte and CoS electrode interface. Nanostructured flower like morphology of CoS craft several ways for charge transportation through its increased interfacial contact which results in low R_ct1_. On the other hand, the flower like morphology of CoS disturbs the interfacial at the grain interior and reduced the charge transportation in it, which is responsible for high R_ct2_. The electron relaxation lifetime was calculated from the peak frequency obtained from the Bode phase plot using the relation τ_e_ = 1/2πf_mid_. Even though, the Warburg ionic diffusion resistance (W_s_) is very low, the electron relaxation lifetime (τ_e_) is found to be high (0.11 s) owing to the presence of high R_ct2_ (50.43 Ω).

#### Asymmetric supercapacitor CoS//AC device

**Figure 9:**
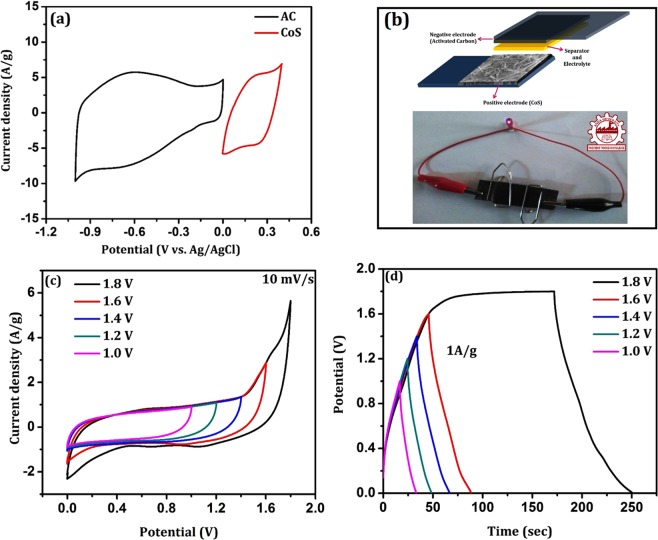
(**a**) CV curve of positive and negative electrode in 3 electrode system at 30 mV/s, (**b**) Photographic image of fabricated asymmetric supercapacitor device, (**c**) CV profile of asymmetric supercapacitor device at different cell voltage and (**d**) charge-discharge profile of asymmetric cell at different cell voltages.

To further demonstrate the electrochemical behavior of prepared electrode material, asymmetric supercapacitor device was fabricated based on pseudo-capacitive hierarchical CoS as positive electrode and commercially available electric double layer capacitive (EDLC) type activated carbon (AC) as negative electrode and 6 M of KOH as supporting electrolyte. In three electrode system, the standard working potential range of CoS was found to be 0 to 0.4 V and the working potential range of AC was between 0 to −1 V (Fig. 9a). Based on the individual working electrode potential in 3-electrode system, the operating cell voltage for the fabricated asymmetric CoS//AC device was optimized as 1.8 V (Fig. 9a). Generally, the advantage of asymmetric supercapacitor device configuration is attaining a high potential window over symmetrical devices. In order to obtain good electrochemical properties of the fabricated asymmetric CoS//AC device, charge balance between 2-electrodes are essential $({q}_{+}={q}_{-})$ which can be obtained by the mass balance using the following relation^35^,

3 $${q}_{+}={C}_{+}X\,{\rm{\Delta }}{V}_{+}X\,{m}_{+}$$

4 $${q}_{-}={C}_{-}X\,{\rm{\Delta }}{V}_{-}X\,{m}_{-}$$

5 $$\frac{{m}_{+}}{{m}_{-}}=\frac{{C}_{-}X\,{\rm{\Delta }}{V}_{-}}{{C}_{+}X\,{\rm{\Delta }}{V}_{+}}$$

where, q_+_ and q_−_ are the charge stored at positive and negative electrode respectively, C represents the specific capacitance (F/g), ΔV corresponds to potential window (V) of the cell and m is the mass of active electrode material (g). The estimated mass of positive electrode material of asymmetric CoS//AC device was 3.2 mg.

The schematic representation of designed asymmetric supercapacitor device and the photographic image of fabricated device were shown in Fig. 9b. The cyclic voltammograms of fabricated asymmetric CoS//AC device at different cell voltage with constant scan rate of 10 mV/s is shown in Fig. 9c. It exhibits quasi-rectangular shaped behaviour which confirms the fast ion transportation and redox reaction kinetics in the device. It may be owing to combined effect of two different charge storage mechanisms (Pseudo-capacitive and EDLC). During the galvanostatic charge-discharge process, K^+^ and OH^−^ ions in the electrolyte move towards negative and positive electrode respectively. Adsorption/desorption of K^+^ ions in the negative electrode instigate the EDLC behaviour in the fabricated device, whereas the surface redox reaction of OH^−^ ions in the positive electrode can form pseudo capacitive behaviour^18^.

Figure 9d shows the galvanostatic charge-discharge (CD) profile of fabricated CoS//AC device for various cell voltage. It reveals triangular shaped charge-discharge curves which illustrate an excellent electrochemical reversibility and Columbic efficiency. The calculated specific capacitances at 1 A/g with corresponding cell voltages of 1 V, 1.2 V, 1.4 V and 1.6 V are 17 F/g, 20 F/g, 25 F/g and 27 F/g respectively (given as Supplementary Information Fig. S7). The maximum specific capacitance (C_s_) of 57 F/g was realized for 1 A/g current density with cell voltage of 1.8 V, while the C_s_ gets reduced with decreasing the cell potential. The charge-discharge profile of CoS//AC asymmetric device at 1 A/g with cell voltage of 1.8 V is anomalous, which is owing to oxygen evolution at high potential window. It resulted in slow charge rate observed from CD profile of CoS//AC device at high cell voltage. In addition, the CV curves and CD profiles of asymmetric CoS//AC device for different cell voltage with various scan rates (10 to 100 mV/s) and current densities (1 to 8 A/g) respectively were shown in Fig. S6 of Supplementary Information. Also, the specific capacitance plot as a function of cell voltage and current density were given in Fig. S7 of Supplementary Information.

**Figure 10:**
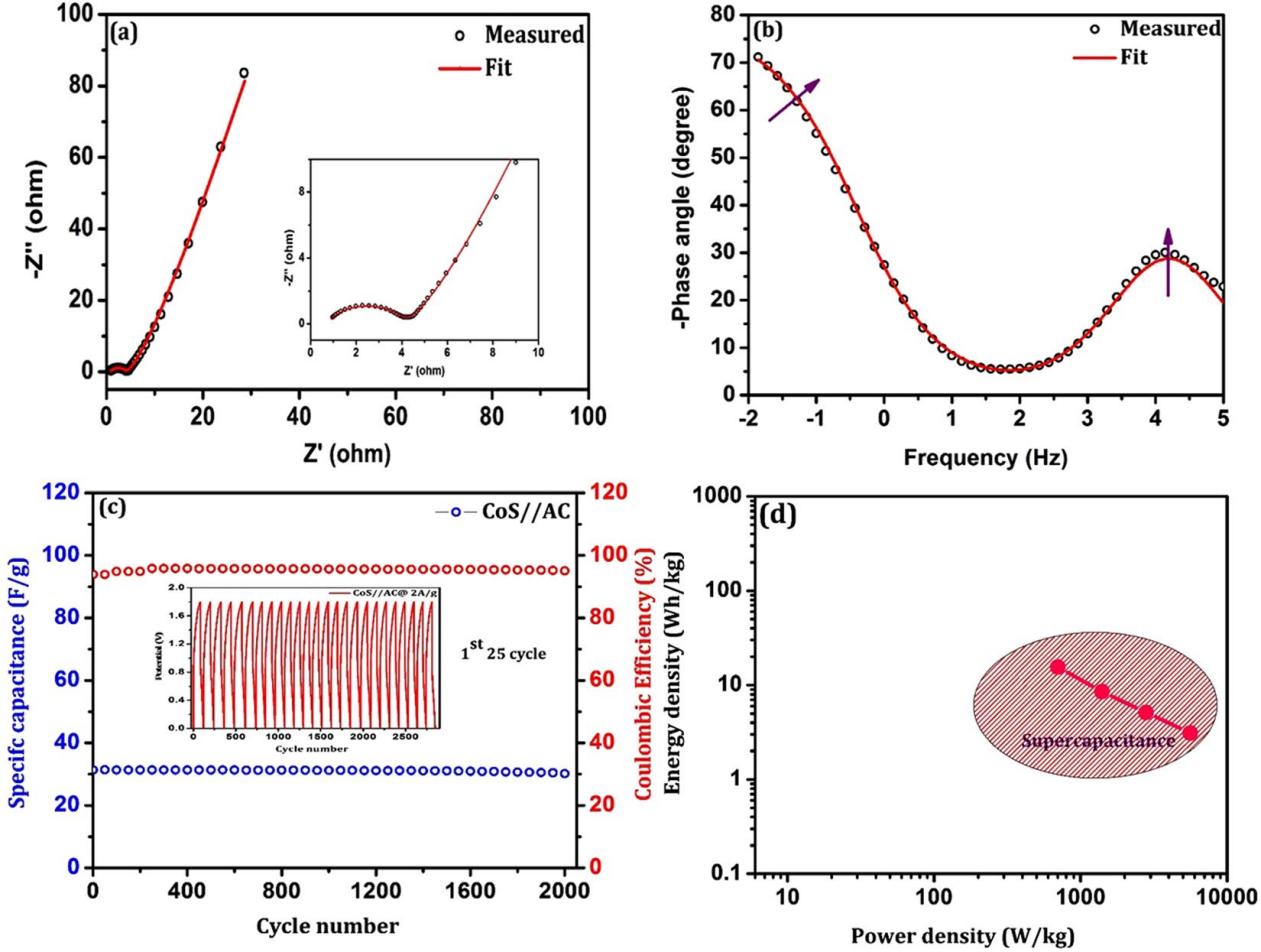
(**a**) EIS-Nyquist plot of asymmetric device at open circuit potential (Inset shows high frequency semicircle), (**b**) Bode phase plot of asymmetric device, (**c**) Specific capacitance and Coulombic efficiency of asymmetric device as a function of cycle number (Inset shows 1^st^ 25 cycle of charge-discharge profile) and (**d**) Ragone plot of fabricated asymmetric supercapacitor device.

Figure 10a shows EIS-Nyquist plot of asymmetric CoS//AC device which is used to evaluate the interfacial charge transport mechanism in 2-electrode system. It reveals two semicircles at high and mid frequency regions respectively. The obtained data was fitted with the same equivalent circuit used in three-electrode system and electrochemical parameters were presented in Table 2, where R_s_ be the series resistance which contributes the surface sheet resistance of current collector (0.69 Ω). Inset of Fig. 10a clearly shows the high frequency semicircle which is attributed to combined effect of charge transport resistances (R_ct1_ = 3.448 Ω) at electrode/electrolyte interfaces (AC/KOH and CoS/KOH)^36^. The mid frequency semicircle is due to charge transport resistance (R_ct2_) at grain interior of CoS based electrode. The linear response at low frequency region making an angle of 45° with the real axis corresponds to low ionic diffusion resistance (W_s_). The presence of double semicircle was further evidenced with observed two phase shifts in Bode phase plot (Fig. 10b). From the peak frequency of the intermediate frequency region, the electron relaxation lifetime was estimated using the relation^28^ τ_e_ = 1/2πf_mid_ and summarized in Table 2. The obtained high τ_e_ of 0.59 s is owing to high R_ct2_ of 79.15 Ω at electrode/electrolyte interfaces. In order to analyze the stability of CoS//AC asymmetric supercapacitor device, galvanostatic charge-discharge measurement was taken over 2000 cycles at constant 2 A/g current density. Figure 10c shows the specific capacitance and Coulombic efficiency of asymmetric device as a function of cycle number. It exhibits that the constructed CoS//AC asymmetric device retains 97.9% of its initial capacitance (31 F/g) after 2000 cycles which confirmed the high cyclic stability. Inset of Fig. 10c shows first 25 cycles of charge-discharge profile of CoS//AC asymmetric device. The Coulombic efficiency of fabricated asymmetric supercapacitor CoS//AC device was retained at 95% after 2000 cycles at current density of 2 A/g (Fig. 10c).

**Table 2:** Electrochemical parameters of fabricated asymmetric device.

Sample	R_s_(Ω)	R_ct1_(Ω)	R_ct2_(Ω)	W_s_ (Ω)	τ(s)	χ^2^
CoS//AC	0.69	3.448	79.15	0.1737	0.59	6.96E-4

A typical Ragone plot of the fabricated CoS//AC asymmetric device for constant cell voltage of 1.8 V with various current densities is shown in Fig. 10d. It depicts the energy density (E_d_) and power density (P_d_) of the constructed device derived from the following relation^37,38^,

6 $$\,{E}_{d}=\frac{\frac{1}{2}C{V}^{2}}{3.6}$$

7 $${P}_{d}=\frac{3600\,X\,{E}_{d}}{{D}_{t}}$$

where, C is the specific capacitance (F/g), V is the cell voltage (V) and D_t_ corresponds to discharge time (s). It can be noted from the plot, the obtained energy and power density of CoS//AC asymmetric device for 1 A/g is 15.58 Wh/kg and 700.12 W/kg respectively. Even at high current density of 8 A/g, the calculated energy-power density values are acceptable (energy density of 3.11 Wh/kg and power density of 5600.7 W/kg) and also in consistent with the previous reported results in Table S2 of Supplementary Information. The obtained power density of prepared CoS//AC asymmetric device is high even at low energy density. Hence, the appreciable electrochemical properties of prepared hierarchical CoS with high cyclic stability and Coulombic efficiency can be extended to commercial applications.

#### J-V characteristics of fabricated DSSCs

**Figure 11:**
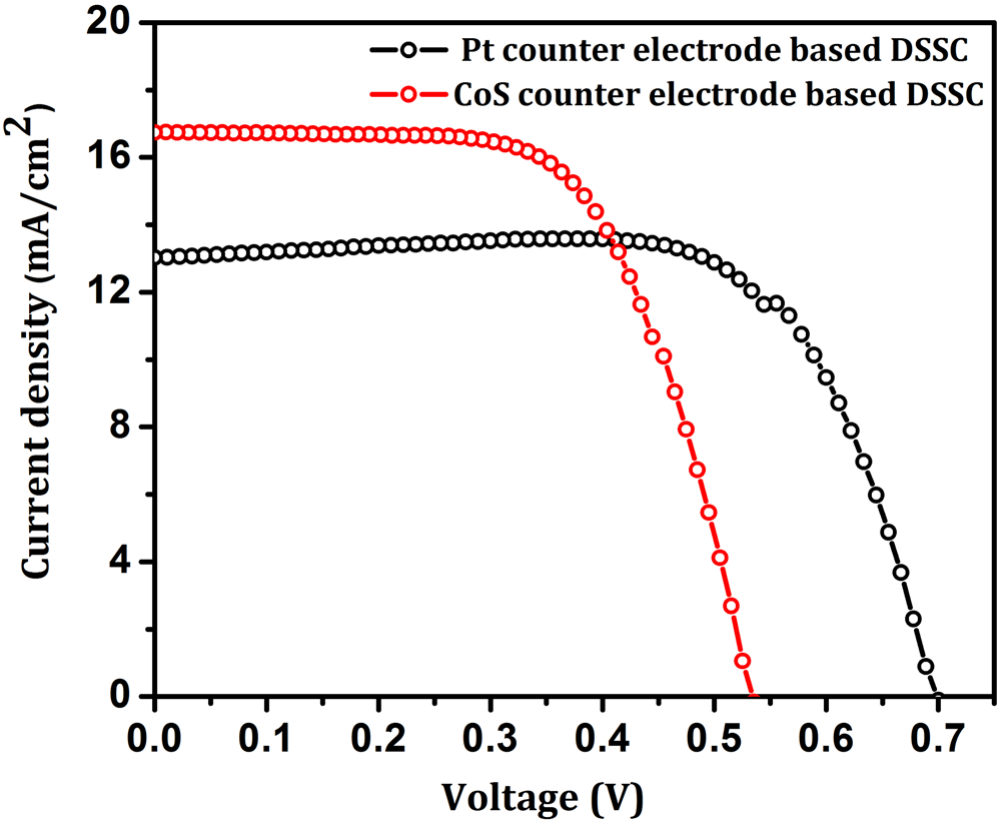
J-V characteristics of fabricated DSSCs.

Figure 11 shows the current-voltage characteristics of DSSCs fabricated using platinum (Pt) and CoS counter electrode. The fabricated DSSCs were tested with standard AAA solar simulator under 1 sun illumination condition (1sun = 0.1 W/cm^2^; AM 1.5 G filter) using Xenon lamp of 450 W. The fill factor (FF) and power conversion efficiency (PCE) of the devices were calculated from the J-V curve using the following relation,

8 $$FF( \% )=\frac{{I}_{max}\,{V}_{max}}{{I}_{sc}\,{V}_{oc}}=\frac{{P}_{max}}{{I}_{sc}{V}_{oc}}$$

9 $$\eta ( \% )=\frac{\,{V}_{oc}{I}_{sc}\,FF\,}{{P}_{in}}$$

where, I_sc_ is short circuit current, V_oc_ is open circuit voltage, FF denotes the fill factor, P_in_ is the total power input and η represent the power conversion efficiency of the device^39^.

The fabricated DSSC based on low cost hierarchical structured CoS counter electrode shows the PCE of 5.704% which is comparable with the efficiency (6.446%) of standard Pt based DSSC (Table 3). It is noteworthy that, the FF and V_oc_ get decreased in CoS based DSSC owing to its reduced transparency which increases the recombination dynamics of the device. Existence of mixed valence of Co (Co^3+^ and Co^2+^) elevates the redox potential of LiI electrolyte^33^ and forced to recombine with conduction band (CB) electrons. In addition, presence of mixed valence of Co ion instigates the intermediate trap levels between conduction and valence band and reduced the V_oc_ of the device. However, the increased J_sc_ is obtained in CoS based counter electrode owing to wide absorption from visible to NIR spectral region (supported with the photoconductivity plot of CoS counter electrode in Fig. S8 of Supplementary Information).

**Table 3:** Photovoltaic parameter of Pt and CoS based DSSCs.

Samples	*J*_sc_(mA/cm^2^)	*V*_oc_(V)	*FF* (%)	η(%)
TiO_2_/N719/LiI/Pt	12.97	0.699	71.10	6.446
TiO_2_/N719/LiI/CoS	16.75	0.535	63.65	5.704

#### EIS-Nyquist plots of fabricated DSSCs

**Figure 12:**
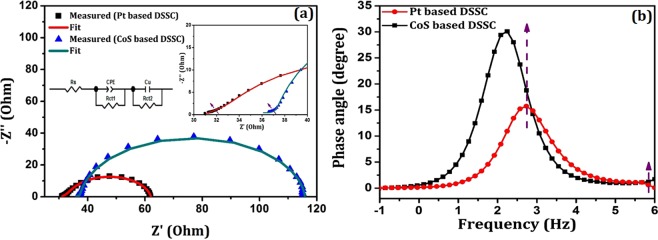
EIS: (**a**) Nyquist plot (Inset reveals high frequency semicircle) and (**b**) Bode phase plot of fabricated DSSCs.

Interfacial electrochemical properties and charge transport phenomena of the fabricated DSSCs were evaluated using Nyquist plots of EIS analysis. Injecting an electrolyte between photoanode and counter electrode, measurements were done in two electrode device configuration (DSSC) for an applied open circuit potential (OCP) in the frequency range of 1 MHz to 0.1 Hz at ambient condition. Figure 12a shows the Nyquist plots of Pt and CoS counter electrode based DSSCs which results in double semicircle behavior. Presence of two semicircles affirms the contribution of two interfacial charge transport resistances in the fabricated devices. Semicircle arc in the high frequency region is attributed to charge transport resistance at counter electrode/LiI electrolyte interface. In the same way, the semicircle at mid frequency regime is owing to charge transport resistance in TiO_2_/N719/LiI electrolyte interface. Intersection of semicircle arc at the real axis of impedance provides the charge transport resistances (R_ct1_ and R_ct2_) at high and mid frequency region respectively. In addition, R_s_ represents the series resistance which contributes to the sheet resistance of FTO and electrolyte diffusion resistance.

The obtained semicircles are fitted with an equivalent circuit model (Inset of Fig. 12a) and the fit data are summarized in Table 4. It can be noted that the interfacial charge transport resistance R_ct1_ of CoS based DSSC (3.89 Ω) is lower than that of Pt based DSSC (4.16 Ω) which is due to increased interfacial contact between CoS and LiI electrolyte. Further, the obtained chemical capacitance (C_µ_) in CoS based DSSC is higher when compared with Pt based device. This is due to wide absorption of CoS from visible to NIR region, which increases the excitation of electrons and results in high electron concentration in CB TiO_2_. In addition, the increase in R_ct2_ is observed for CoS counter electrode based device, owing to the mixed valence state of Co^28^. It upsurges the redox potential of LiI electrolyte and therefore the recombination dynamics between CB electrons and LiI gets increased which is responsible for the reduced FF in CoS based DSSC in J-V measurements.

**Table 4:** Electrochemical parameters and charge transport kinetics of Pt and CoS counter electrode based DSSCs.

Samples	R_s_ (Ω)	R_ct1_ (Ω)	τ_t_ (ms)	R_ct2_(Ω)	C_µ_ (F)	τ_e_ (ms)	D_e_ X 10^−2^ (cm^2^/s)	L_n_ (μm)	Φ_c_ (%)	χ^2^ x 10^−5^
Pt based DSSC	30.83	4.16	0.113	27.12	2.723E-5	0.622	5.7391	59.75	81.78	2.96
CoS based DSSC	34.07	3.89	0.117	77.96	2.997E-5	2.798	4.5725	113.11	95.82	20.9

The presence of two interfacial charge transport resistance can be further confirmed from the phase angle shift in the Bode phase plots (Fig. 12b). The electron relaxation lifetime (τ_e_) was calculated from the peak frequency (f_max_) of the Bode phase plots using the relation,

10 $${\tau }_{e}=\frac{1}{{\omega }_{max}}=\frac{1}{2\pi {f}_{max}}$$

It is noteworthy that the obtained electron relaxation lifetime of Pt based DSSC (0.622 ms) is lower than CoS based device (2.798 ms). Higher τ_e_ of CoS based DSSC resulted to have longer relaxation lifetime of electrons in CB TiO_2_ and hence induces higher recombination possibility in the device (which is responsible for low FF). In addition, the charge transport lifetime (τ_t_), diffusion co-efficient (D_e_), diffusion length (L_n_) and charge collection efficiency (ϕ_c_) were also calculated using the following relation:

11 $${{\rm{\tau }}}_{{\rm{t}}}={{\rm{R}}}_{{\rm{ct1}}}{{\rm{C}}}_{\mu }$$

12 $${{\rm{D}}}_{{\rm{e}}}={{\rm{L}}}^{{\rm{2}}}\,/\,{{\rm{R}}}_{{\rm{ct1}}}{{\rm{C}}}_{\mu }$$

13 $${{\rm{L}}}_{{\rm{n}}}={({{\rm{D}}}_{{\rm{e}}}{{\rm{\tau }}}_{{\rm{e}}})}^{1/2}$$

14 $${{\rm{\Phi }}}_{{\rm{c}}}={\rm{1}}-{({\rm{\tau }}}_{{\rm{t}}}\,/\,{{\rm{\tau }}}_{{\rm{e}}})$$

Though, the obtained diffusion length and charge collection efficiency is low in Pt based device, its PCE is higher than CoS based DSSC owing to its low charge transport resistance (R_ct2_) and short electron relaxation lifetime (τ_e_). On the other hand, the high charge collection efficiency of CoS based DSSC evidences the increased J_sc_ value of 16.75 mA/cm^2^.

#### Magnetic and Electrical characterization

**Figure 13:**
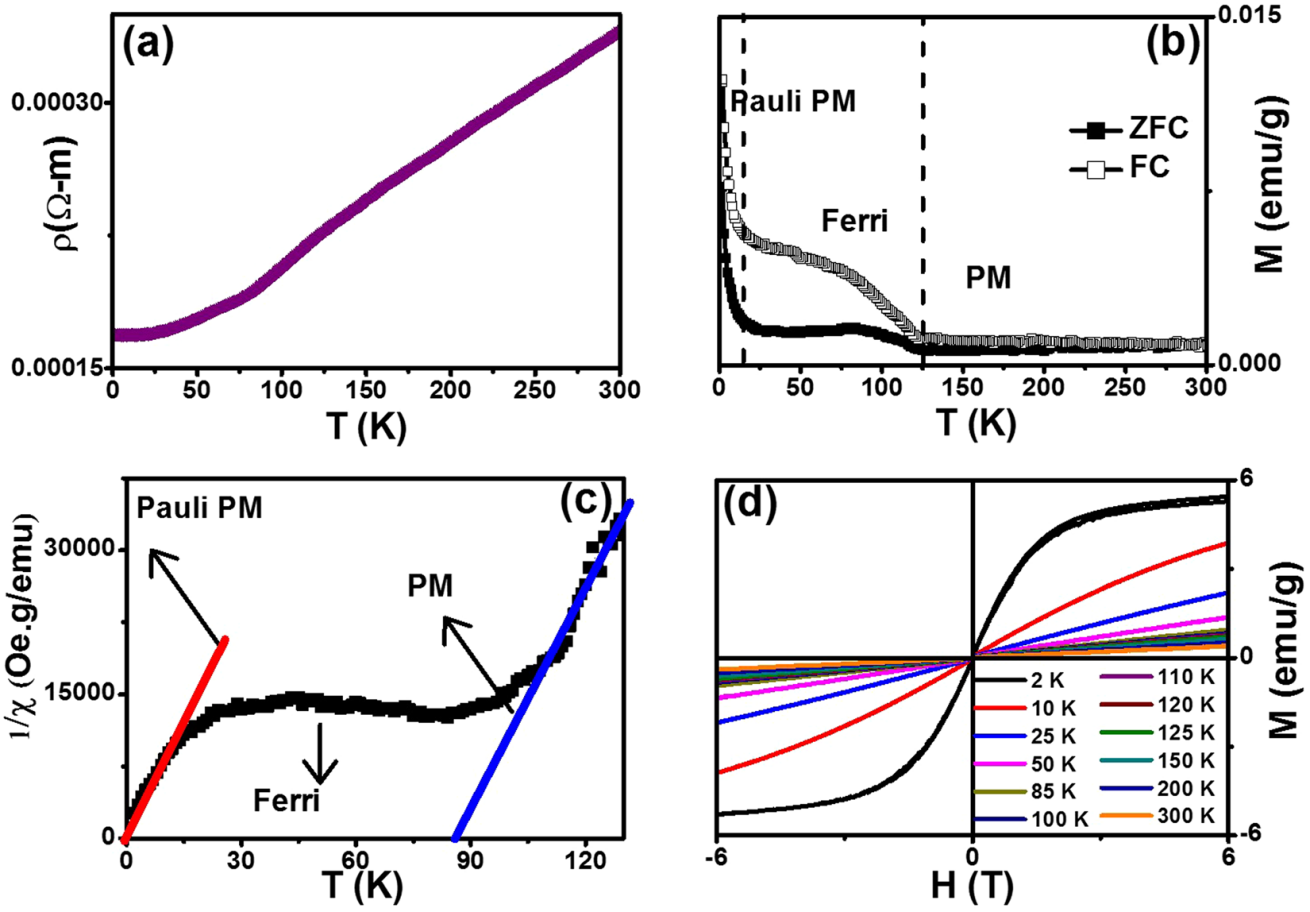
(**a**) Temperature dependent resistivity of CoS without any magnetic field, (**b**) Temperature dependent magnetization of CoS at constant field of 20 Oe during zero field cooling (ZFC) and field cooling (FC) cycles, (**c**) Curie-Weiss Plot for ZFC Cycle and (**d**) M-H loop with Coercive field and Remanent magnetization as a function of temperature.

Figure 13a shows the temperature dependent resistivity of CoS without any applied magnetic field. While reducing the temperature from room temperature (300 K), there is a linear decrement in the resistivity till 20 K which infers the presence of metallic nature. Interfacial contact in the hierarchical flower shaped CoS nanoparticles is adequate for electron hopping through its tightly bounded grains which exhibit metallic nature owing to electron transport between the mixed valence states of cobalt. Further reduction in the temperature exhibiting the semiconducting nature of CoS. The transition of metallic to semiconducting nature in our sample could be explained based on Peierls transition^40^ as follows: In the present study, the grain size of CoS nanoparticles is 46 nm which could behave as 1 dimensional (1-D) system. The electronic configuration of Co^2+^ is [Ar] 3d^7^ 4s^2^, whereas the electronic configuration of S^2−^ is [Ne] 3s² 3p^4^. As the 2 valence electrons of cobalt compensate the sulfur vacancy, a hole remains in the d state of cobalt. On the other hand, presence of Co_3_S_4_ defect (confirmed by XRD and XPS) affirmed the charge imbalance which uses the existing hole in the cobalt to behave as metallic nature, until thermal fluctuations are sufficiently large enough. When the temperature is low (<20 K), atoms are getting closed and subsequently, the periodicity of the system is now behaved as two dimensional (2-D) system. Hence, the electrons will not stay at their lattice sites, but to only vibrate with each other. The vibrational distance between two electrons will differ. i.e., alternating shorter and longer owing to the dimensional change and finds a definite energy gap in the spectrum of electrons. It accounts for certain amount of energy to push the electrons out of this state. As a consequence of this transition, the system is no longer a conducting one, but rather semiconducting at a very low temperature region (<20 K).

Figure 13b shows the temperature dependent magnetization of CoS nanoparticles at a constant magnetic field of 20 Oe during ZFC and FC cycles. While reducing the temperature, the sample has very low magnetization value till 120 K, and behaves identical during both ZFC and FC cycles. The effective magneton on this region has been estimated, and the value is found to be 0.0841 μ_B_ which affirms the presence of paramagnetic nature. Below 120 K, there is a sudden increase in magnetization, that may suggests the existence of ferromagnetic (or) ferrimagnetic transition. From the Curie-Weiss plot of ZFC curve (Fig. 13c), the analogue for this transition is slightly raised rather being sharp and this trend confirms the presence of ferrimagnetic nature in CoS. The ferrimagnetic transition temperature is determined to be 85 K from Curie-Weiss plot (blue slanting line in Fig. 13c). When the temperature gets reduced further, the magnetization shows sharp rise below 20 K which passes through the origin in Curie-Weiss plot (red slanting line in Fig. 13c) suggesting the appearance of Pauli-paramagnetic region^41^. It is also further confirmed by having the small effective magnetic moment (0.096 μ_B_) in this region. Thus, the transition of ferrimagnetic to Pauli paramagnetic transition is an analogue of metallic to semiconducting transition in the prepared sample.

Figure 13d shows the field dependent magnetization at various temperature points in the four quadrants of magnetic field upto 6 T. From 300 K to 85 K, the hysteresis loops (M-H) shows a perfect straight lines till 6 T, confirming the paramagnetic nature of prepared CoS. The M-H plots at 85 K and 50 K also displays straight lines of magnetization, where the rate of increase in magnetization with the field is comparatively higher than that of the previous temperature points. Further examination of the M-H plot at 25 K has slight bending in its path, suggesting the ferrimagnetic nature around this temperature. On the other hand, M-H loop at 2 K shows the straight line upto 2 T due to Pauli paramagnetic nature at a very low magnetic field. However, the magnetization gets almost saturated above 2 T signifying the possibility of ferromagnetic transition at higher magnetic fields. It might be possible as the high magnetic field can align all the dipoles of Co^3+^, Co^2+^ and S^2−^ in a particular directions and could be the significance for ferromagnetic nature of the CoS.

## Conclusions

In summary, hierarchical nanostructured CoS was prepared using facile hydrothermal reaction and its structural, optical, elemental, electrical, morphological properties were well explored. X-ray diffraction result confirmed the formation of hexagonal CoS. Presence of additional cubic Co_3_S_4_ phase originated the mixed valence state was affirmed by XPS analysis. HRSEM images of CoS showed hierarchical star anise flower like morphology. Enhancement in the electrochemical properties by the mixed valence state of cobalt can be effectively used for the applications of dye-sensitized solar cells and supercapacitors. Electrochemical properties of prepared CoS were characterized using two and three electrode systems and their supercapacitive performances were examined. It exhibited maximum specific capacitances of 57 F/g in 2-electrode system and 348 F/g in 3-electrode configuration for 1 A/g. An appreciable energy density of 15.58 Wh/kg and power density of 700.12 W/kg were obtained at 1 A/g for the fabricated CoS//AC asymmetric supercapacitor device. Cyclic stability measurement of fabricated device retains 97.9% of capacitance after 2000 cycle with high Coulombic efficiency (95%). In addition, the PCE of 5.7% was realized in prepared CoS based counter electrode in DSSC application. The interfacial charge transport kinetics such as charge transport resistances, chemical capacitance, electron relaxation lifetime, diffusion co-efficient, diffusion length and charge collection efficiency were evaluated by Nyquist plot of electrochemical impedance spectroscopy. In addition, the temperature dependent magnetic properties of the prepared CoS reveals paramagnetic to ferrimagnetic transition at 85 K and ferrimagnetic to Pauli-paramagnetic transition at 20 K respectively as evidenced from Curie-Weiss plot. The metallic nature of CoS has low resistivity by its efficient electron transportation between the mixed valence states of cobalt. Based on the Peierl’s transition effect, the metallic nature gets transformed to semiconducting behavior <20 K.

## Experimental Methods

### Materials

Reagent grade chemicals of CoCl_2_.6H_2_O and CH_4_N_2_S were purchased from Sigma Aldrich-India for the preparation of nanostructured CoS. For the fabrication of asymmetric supercapacitor activated carbon (AC), carbon felt and KOH were purchased from Alfa Aesar, India. TiO_2_ paste (P25-Degussa), N719 dye [Di-tetrabutylammonium cis-bis (isothiocyanato) bis(2,2′-bipyridyl-4,4′-dicarboxylato) ruthenium(II)] and FTO substrate (~7 Ω/cm^2^) of Sigma Aldrich were purchased for the construction of dye-sensitized solar cells. Standard platinum counter electrode and LiI electrolyte were procured from Dyesol, Australia for the fabrication of DSSC.

### Synthesis of Hierarchical CoS nanoflower

The hierarchical CoS star anise like architecture was prepared by facile one step hydrothermal method. In a typical preparation procedure of CoS, cobalt chloride hexahydrate (CoCl_2_.6H_2_O) (0.4 M) and thiourea (CH_4_N_2_S) (1.2 M) were dissolved in 30 ml of distilled water using magnetic stirring for 30 min. Then the solution was transferred into 75 ml of Teflon lined stainless steel autoclave for hydrothermal treatment at 180 °C for 24 hours. After cooling down to room temperature, the precipitate was collected and washed with ethanol and distilled water for several times and dried in air at 60 °C.

### Characterization techniques

#### Materials characterization

For structural confirmation, X-ray diffraction (XRD) pattern of CoS was obtained by X-ray powder diffractometer (SEIFERT JSO-2002) using Cu–K_α1_ radiation with operating voltage at 30.0 kV and analyzed by XRDA 3.1 software. Fourier transform infrared spectrum of CoS was studied using FTIR-Perkin Elmer Spectrum_1_ for functional group analysis. Surface morphology of prepared sample was examined with high-resolution scanning electron microscope (HRSEM; Hitachi S4800). The optical absorption spectrum of CoS was measured using UV-Visible spectrometer in diffused reflectance spectra (DRS mode) using Perkin Elmer-Lamda 45. The valence state and surface chemical composition of CoS was analysed using X-ray photoelectron spectroscopy (XPS) with dual Al/Mg anodes as X-ray source (Thermo Fisher Scientific, USA) and the base pressure of the instrument was 5.0 × 10^−10^ mbar. Film thickness of prepared CoS counter electrode and TiO_2_ photoanode were calculated from the surface profiler (Surf Test SJ-210).

#### Electrochemical characterization

The electrochemical properties such as cyclic voltammetry (CV) and galvanostatic charge-discharge profile (GCD) of prepared CoS was studied using electrochemical work station (Autolab-PGSTAT-302N) under two and three electrode configuration system with 6 M KOH as supporting electrolyte. The interfacial charge transport properties of fabricated DSSCs and asymmetric supercapacitor in two electrode device configuration were also evaluated using EIS-Nyquist plot. The power conversion efficiency of fabricated DSSCs were calculated from the J-V characterization measured by AAA-Solar simulator (Oriel-Newport AAA solar simulator) at standard test condition (1 sun intensity = 0.1 mW/cm^2^ with 1.5 G filter) using 450 W xenon lamp. Further, the photoconductivity measurements were done for CoS electrode at optimized visible and NIR lamp illumination using Keithley 6487 voltage source/picoammeter.

#### Magnetic and transport characterization

The temperature dependent magnetic behavior of the prepared CoS was performed using physical property measurement system with the option of vibrating sample magnetometer (PPMS-VSM) (Quantum Design, USA). The temperature dependent zero-field cooling (ZFC) and field cooling (FC) magnetization curves were measured as follow: The as-prepared sample was first cooled in zero field from 300 to 2 K, then the magnetic field (H) was applied. After the ZFC, the magnetic moment (M) was recorded as the temperature increases from 2 to 300 K. Subsequently, the sample was cooled to 2 K again with the constant applied field and the FC magnetic moment (M) was measured by heating the sample to 300 K again in the same field. Electrical resistivity measurement was carried out using closed cycle refrigerator-variable temperature inserts (CCR-VTI). For the conventional four-probe measurement system, the contacts were made by a high-quality silver paste with a copper wire of Φ = 0.15 mm.

### Device Fabrication

#### Fabrication of asymmetric CoS//AC supercapacitor device

Carbon felt was used as current collector for the fabrication of asymmetric supercapacitors. 80 wt% of prepared CoS material, 15 wt% of acetylene black and 5 wt% of polyvinylidene fluoride (PVDF) binder were mixed together with N-Methyl-2-pyrrolidone (NMP) as solvent and ground for 1 hour using agate mortar. Then, the mixed paste was coated on current collector employing the doctor blade technique. After air drying at 80 °C for 2 hours the mass loading of positive electrode material was measured to be 3.2 mg. In the same way, activated carbon (AC) was loaded in another carbon felt as negative electrode material. For charge balance, the mass loaded in negative electrode should be optimized. Finally, the cellulose paper was dipped in prepared 6 M KOH solution and sandwiched between the positive and negative electrodes for the fabrication of asymmetric supercapacitor device.

#### Fabrication of DSSCs

Fluorinated tin oxide (FTO) glass substrates with dimensions of 15 × 15 mm^2^ were taken. For photoanode application, active area of 4 × 4 mm^2^ was defined by masking the FTO substrate using scotch tape. Then, TiO_2_ paste (P25-Degussa) was coated on FTO glass substrate using doctor blade technique and annealed at 450 °C to remove the binder. Later, the prepared TiO_2_ coated (thickness ranging from 23–25 μm) FTO was sensitized in 0.3 mM of ethanolic N719 dye solution for 12 hours. And for the counter electrode, the hierarchical CoS was directly grown on the FTO substrate while preparing the sample in autoclave reactor with the thickness ranging of 26.46 μm (Fig. S1 of Supplementary Information). Finally, dye-sensitized solar cell was fabricated using prepared TiO_2_ based photoanode and CoS based counter electrode by injecting LiI electrolyte in between them.

## Supplementary information

Effect of Bi-functional Hierarchical Flower-like CoS Nanostructure on its Interfacial Charge Transport Kinetics, Magnetic and Electrochemical Behaviors for Supercapacitor and DSSC Applications

## Data Availability

All data generated or analyzed during this study are included in this published article (and its Supplementary Information Files).
