# Supplementary material for: Effect of Bi-functional Hierarchical Flower-like CoS Nanostructure on its Interfacial Charge Transport Kinetics, Magnetic and Electrochemical Behaviors for Supercapacitor and DSSC Applications
Source: Sci Rep. 2019 Feb 4;9:1228. doi: 10.1038/s41598-018-37463-0 (PMC6361894; doi:10.1038/s41598-018-37463-0)
Supplement: Supplementary file 1 — Effect of Bi-functional Hierarchical Flower-like CoS Nanostructure on its Interfacial Charge Transport Kinetics, Magnetic and Electrochemical Behaviors for Supercapacitor and DSSC Applications [file 41598_2018_37463_MOESM1_ESM.pdf]

Supplementary Information

Effect of Bi-functional Hierarchical Flower-like CoS Nanostructure on its Interfacial Charge Transport Kinetics, Magnetic and Electrochemical Behaviors for Supercapacitor and DSSC Applications

K. Ashok Kumar[1], A. Pandurangan[1,*], S. Arumugam[2] and M. Sathiskumar[2]

[1]Department of Chemistry, Anna University, Chennai-600025, Tamil Nadu, India

[2]Centre for High Pressure Research, School of Physics, Bharathidasan University, Tiruchirappalli-620024, Tamil Nadu, India.

*Corresponding author Email id: pandurangan_a@yahoo.com

Legends to Supplementary Figures and Tables:

Additional studies related to thickness measurement of CoS counter electrode and TiO_2 based photoanode were characterized by surface profiler which is given as supplementary information Fig.S1. To evaluate strain induced broadening of X-ray diffraction peaks, Williamson-Hall plot was given in supplementary information Fig.S2. To investigate the repeatability on the formation of CoS star anise morphology, CoS was prepared for the 2nd time and examined with HRSEM analysis which is shown in Fig.S3. Also, the functional group analysis of the prepared CoS was carried out using FTIR measurements and given as Fig.S4 of supporting information. For asymmetric supercapacitor device fabrication, electrochemical studies on negative electrode material (activated carbon) is essential which are presented in Fig.S5-S7 of supplementary information. Further, photoconductivity measurements were given as supporting data (Fig.S8 and Table.S1) for conductivity measurements and NIR absorption of CoS. The literature survey on the usage of CoS nanostructures for supercapacitor application were also presented in Table.S2 of supplementary information.

Thickness measurement using surface profiler:

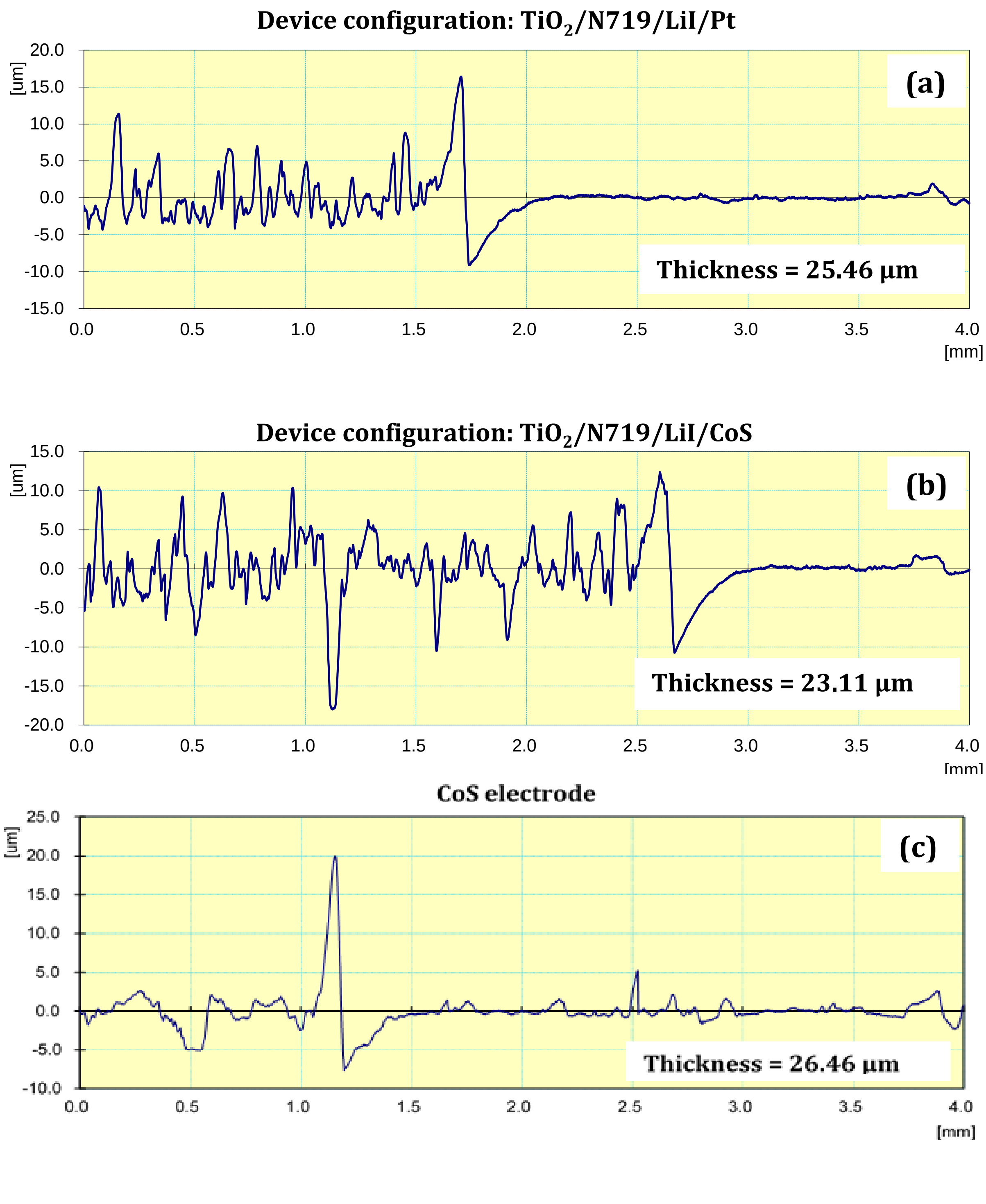

Fig.S1. Thickness data of (a,b) prepared TiO_2 photo anodes and (c) as grown CoS film

Strain plot: Williamson-Hall (W-H) plot:

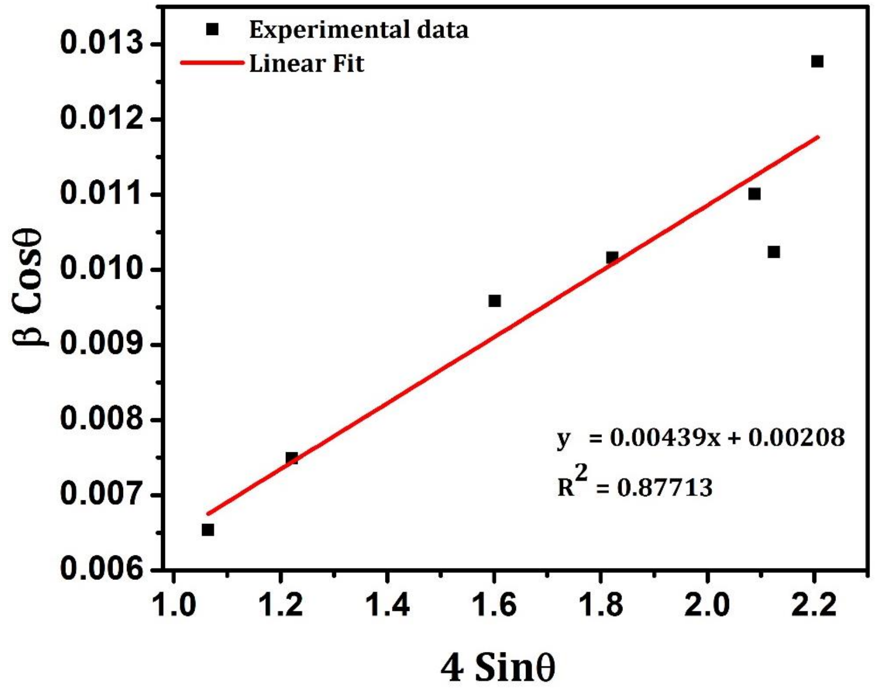

Fig.S2. Williamson-Hall (W-H) plot of prepared CoS

X-ray diffraction peak broadening may arise by three factors such as instrument error broadening, size dependent broadening and lattice strain induced broadening. The instrument error was calibrated by subtracting the line broadening of standard silicon material[1] using the following relation,

$$\beta_{hkl} = [\beta^2_{Measured} - \beta^2_{Instrument}]$$

The possible peak broadening after the instrument error calibration is only due to size and strain. Now, the actual broadening obtained in XRD pattern can be given by the relation,

$$\beta_{hkl} = \frac{K\lambda}{D\mathrm{Cos}\theta} + 4\varepsilon \tan\theta$$

Rearranging the above equation,

$$\mathrm{Cos}\theta\, \beta_{hkl} = \frac{K\lambda}{D} + 4\varepsilon \,\mathrm{Sin}\,\theta$$

The above equation is called Williamson-Hall relation for estimating a micro-strain[2,3]. Assuming that the induced strain is uniform in all crystallographic directions, W-H plot is derived to

calculate the induced lattice strain, where βcosθ is plotted against 4sinθ as shown in Fig.S2. The slope of linear fit provides a lattice strain and Y-intercept estimates the crystallite size of the sample. The obtained strain value of 0.00439 is originated by the presence of cubic Co_3S_4 phase.

Morphological analysis:HRSEM

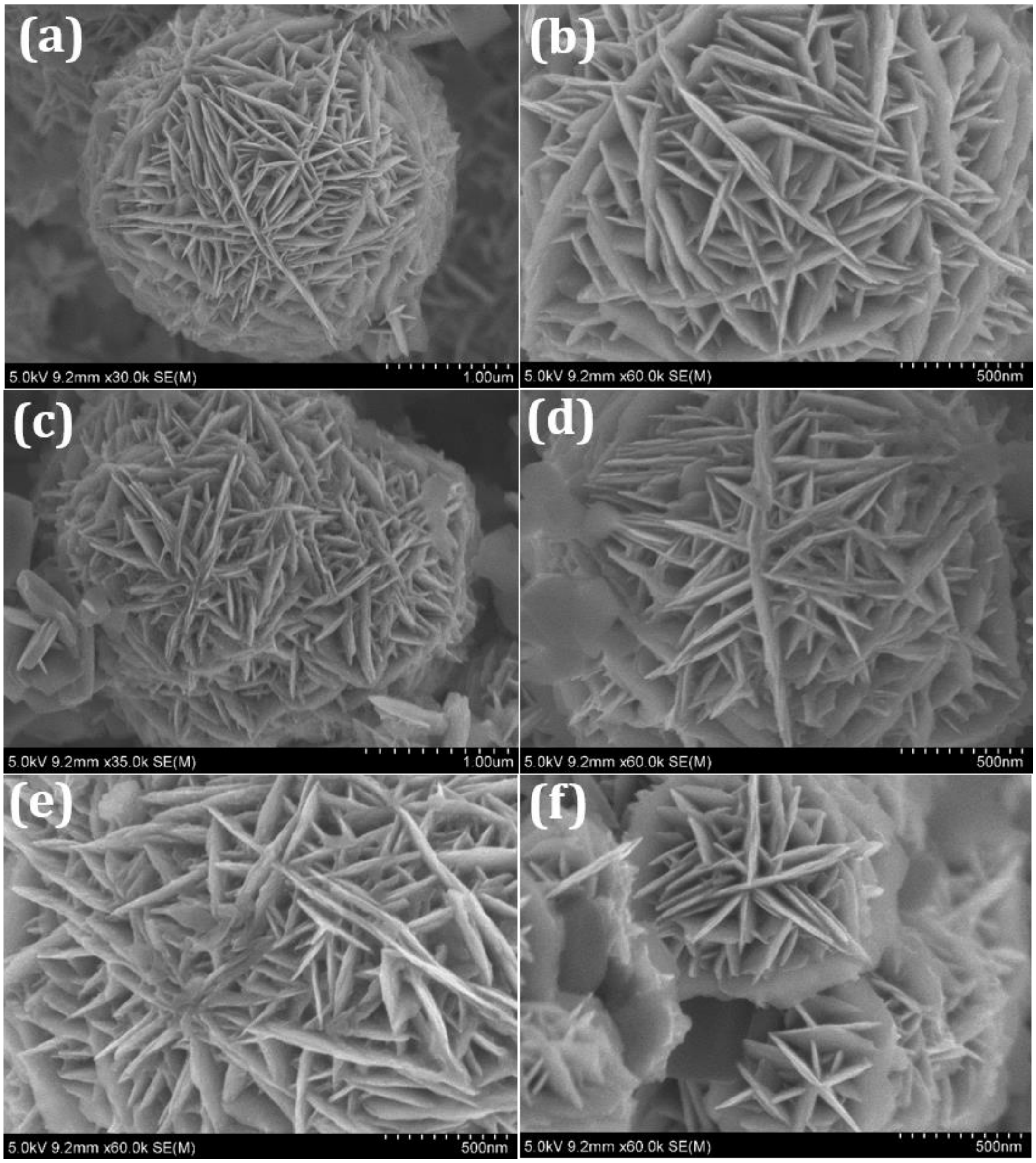

Fig.S3 (a-f) HRSEM images of 2nd time prepared hierarchical flower shaped CoS

Functional group analysis: FTIR

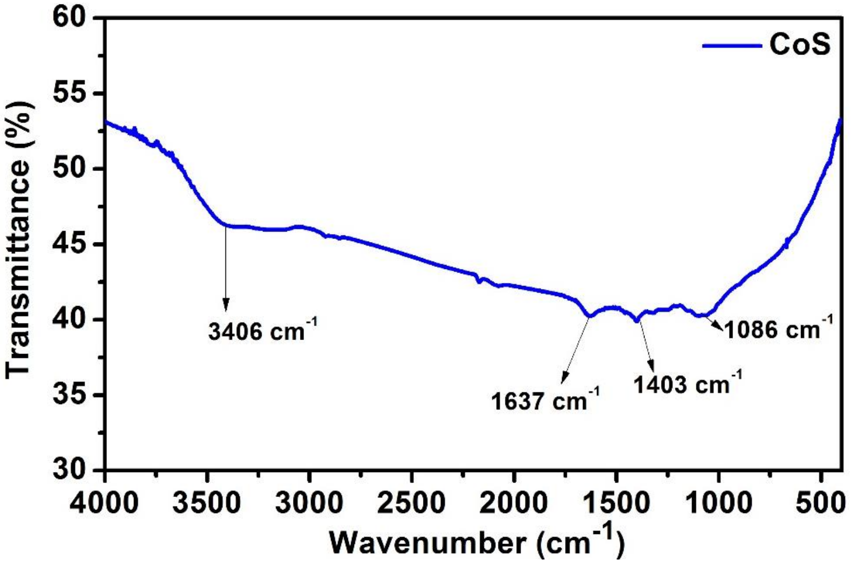

Fig.S4. FTIR spectrum of CoS

The presence of functional groups in the prepared CoS was analyzed using FTIR spectroscopy in the range of 4000 to 400 cm^{-1} shown in Fig.S4. It is noted that the band appeared at 1086 cm^{-1} corresponds to bending vibration of sulfur and cobalt linkage (Co-S) in the prepared sample[4]. The small band appeared at 1403 cm^{-1} and 1637 cm^{-1} are attributed to -OH deformation vibration of Co-OH group[5] and bending vibration of surface adsorbed water (H_2O) molecules respectively. Here, Co-OH was formed by hydrolysis of $CoCl_2.6H_2O$ in hydrothermal reactor. The broad vibrational peak appeared at 3406 cm^{-1} is due to hydroxyl (-OH) bonding on the surface of CoS.

Electrochemical analysis: Cyclic voltammetry and Galvanostatic Charge-discharge profile

Negative electrode materials:

Investigation on positive and negative electrode materials under same electrochemical condition is essential to analyze the performance of fabricated supercapacitor devices. Carbon based materials are extensively used as negative electrode materials for the fabrication of asymmetric supercapacitor devices. In the present study, commercially purchased activated carbon (AC) was employed as negative electrode material and its electrochemical properties were examined using CV and GCD analysis. Measurements were carried out in 3 electrode

configuration using 6M KOH as supporting electrolyte in the potential range of 0 to -1 V for different scan rates and current densities.

Fig.S5a shows the CV curves of AC based electrode, which clearly illustrates the rectangular shaped behaviour and thus the capacitive nature obtained here is instigated from EDLC process. The galvanostatic charge-discharge curve of AC based electrode (Fig.S5b) exhibits triangle shaped CD loop which also affirmed the EDLC behaviour. In addition, the presence of iR drop in GCD profile is caused by poor electrical contact between the electrode material and current collector[4]. From the discharge time, the estimated specific capacitance at 1 A/g, 2 A/g, 4 A/g, 8 A/g are 75 F/g, 44 F/g, 36 F/g and 34 F/g respectively (Fig.S5c).

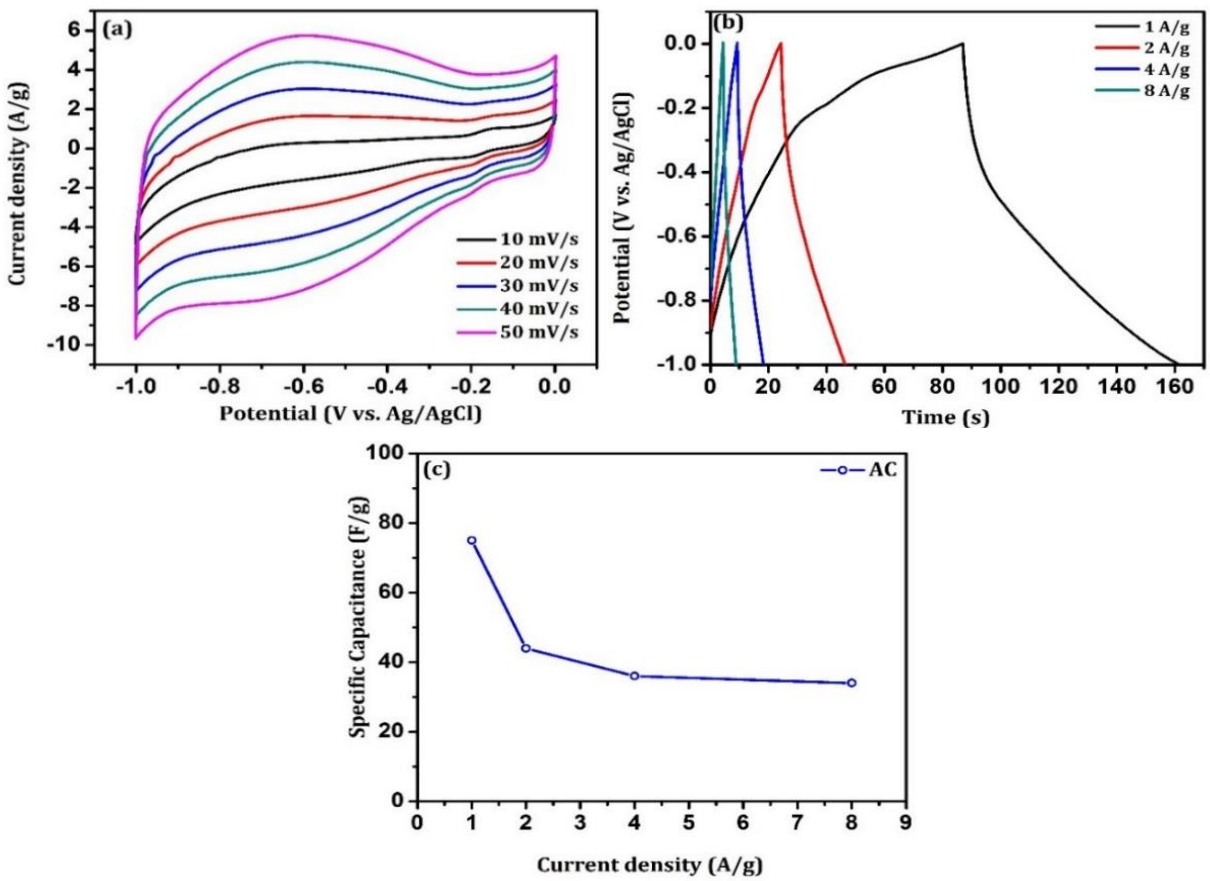

Fig.S5 (a) CV curve of activated carbon at different scan rate, (b) Charge-discharge profile of activated carbon at various current densities and (c) Specific capacitance of activated carbon as a function of current density

CV and CD profile of Asymmetric Supercapacitor device:

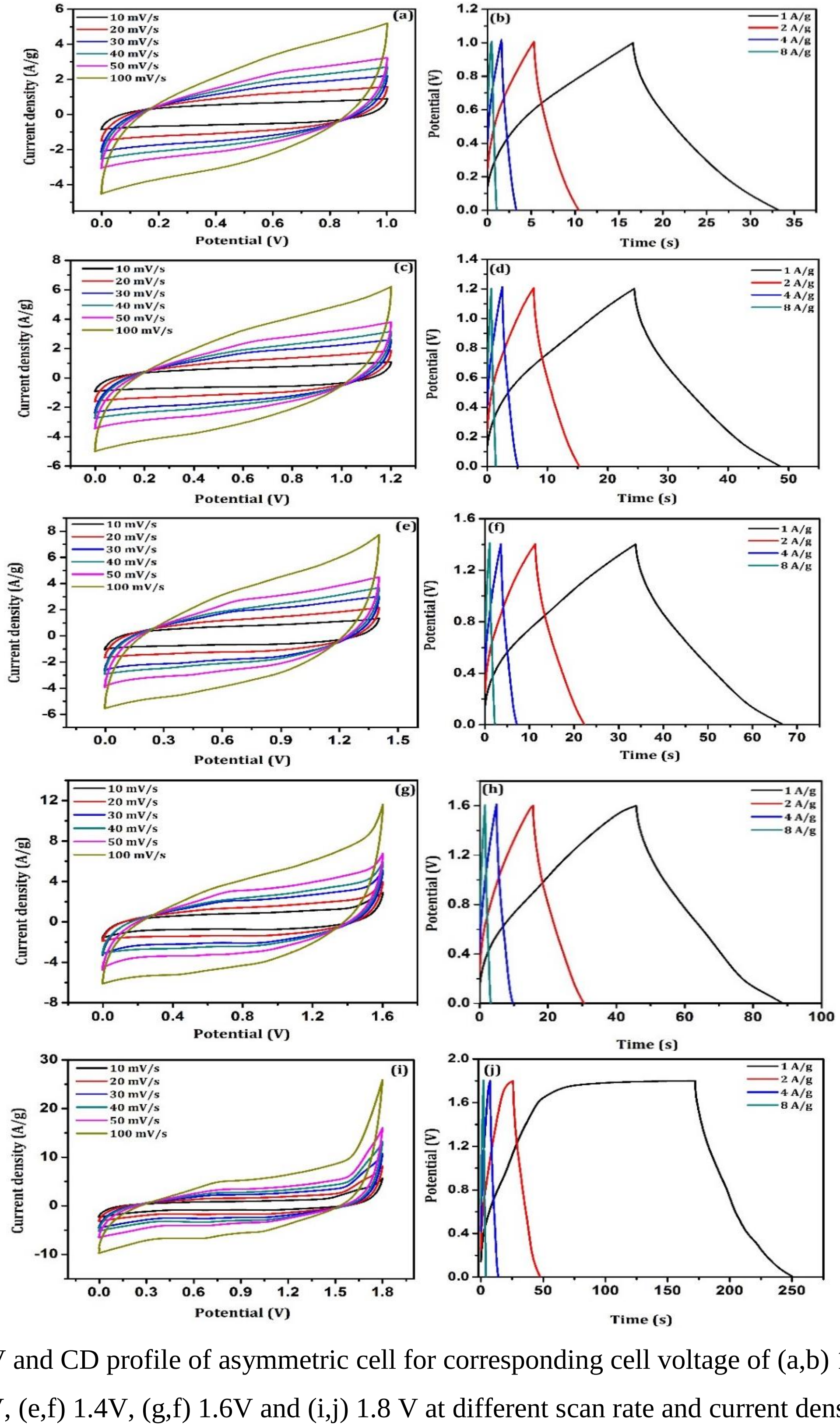

Fig.S6 CV and CD profile of asymmetric cell for corresponding cell voltage of (a,b) 1.0V, (c,d) 1.2V, (e,f) 1.4V, (g,f) 1.6V and (i,j) 1.8 V at different scan rate and current densities

Specific Capacitance as a function of Current density and Cell Voltage:

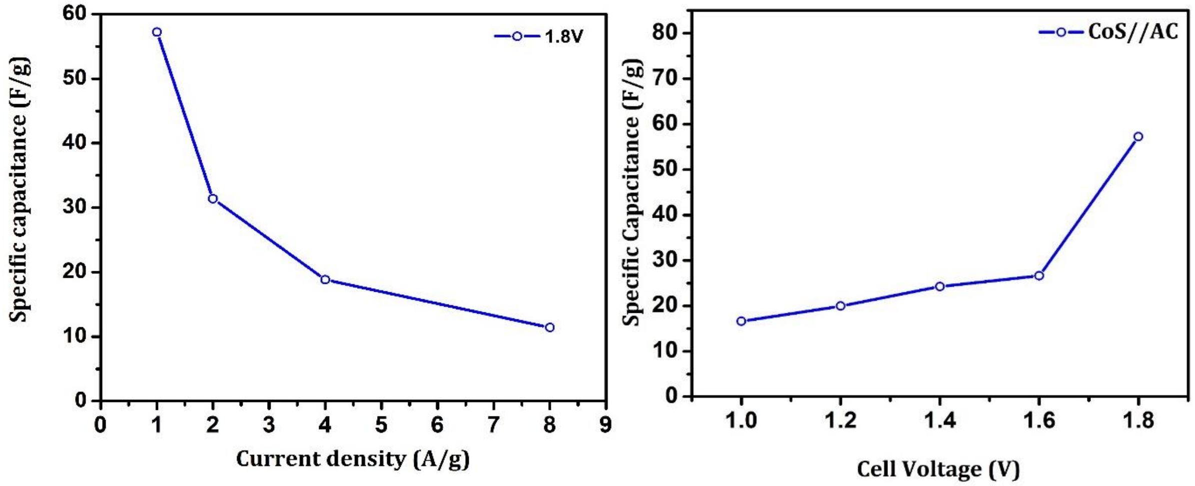

Fig. S7. Specific capacitance of fabricated asymmetric supercapacitor device as a function of (a) Current density and (b) Cell voltage

Photoconductivity studies:

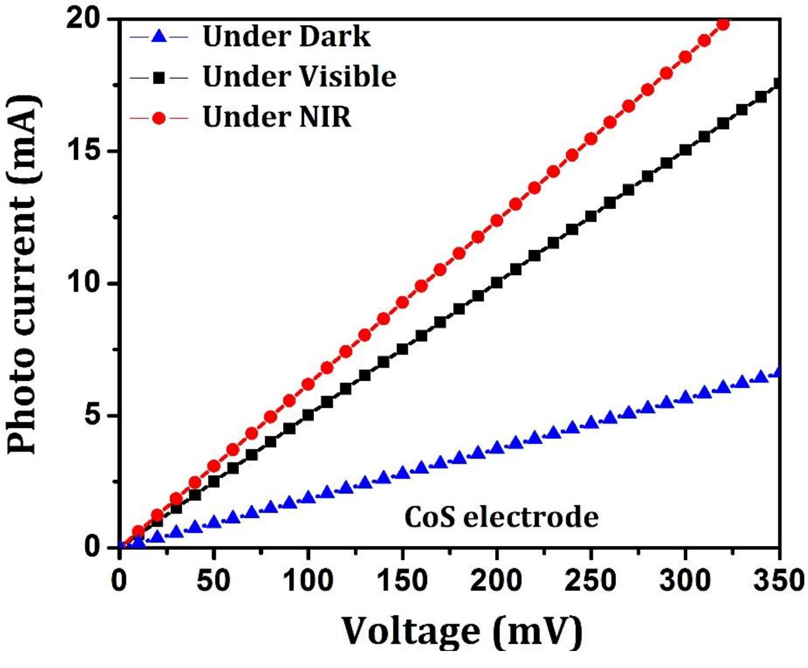

Fig.S8. Photoconductivity measurements of CoS electrode

Table.S1 Conductivity data of hierarchical nanostructured CoS electrode

Samples	Thickness (μm)	Condition	Conductivity (mS/cm)
**CoS electrode**	27.68	Dark	0.0137
		Visible	0.0347
		Near-infrared	0.0428

Fig.S8 shows the photoconductivity plot (I-V curve) of active CoS electrode. Measurements were done using Keithley source meter (6487 pico-ammeter) with the white light (LED) and NIR lamps. Lux meter was utilized for the purpose of calibrating the lamp intensity. The conductivity of prepared CoS electrode under dark and illumination of visible and NIR light is calculated using the relation ($\sigma = l/RA$) and summarized in Table.S1, where σ represents the photoconductivity, R be the resistance (slope of the linear fit) and l is thickness of the CoS film. It is noted that photoconductivity of CoS electrode under visible and NIR illumination condition is higher compared to dark condition. Especially under NIR illumination the conductivity gets increased owing to the NIR light photon absorption of CoS electrode as evidenced with the DRS spectrum of prepared CoS nanoparticles. While using CoS electrode as counter electrode in DSSC, the current density gets increased owing to its wide range of absorption from visible to NIR region. Increased current density will force to do rapid recombination of minority charge carriers with the majority charge carriers[6] and hence the FF of CoS based DSSC gets decreased.

Table.S2. Literature survey on CoS nanostructures for supercapacitor application

Sample	Synthesis technique	Morphology	Specific capacitance (F/g)	Current density (A/g)	E_d Wh/kg	P_d W/kg	Cycle Number	Capacitance Retention (%)	Ref
**CoS**	Microwave	Nanoprism	224	1	---	---	1000	97	7
**CoS**	Solvothermal	Nanoprism	156	1	---	---	---	---	7
**CoS_2**	Hydrothermal	Octahedran	237	1	11.8	300	2000	92.6	8
**CoS**	Hydrothermal	Nanotubes	285	0.5	14.25-4.8	150-3000	1000	86.5	5
**CoS**	Recycling method	CoS hierarchitecture	409	1	---	---	1000	94.8	9
**CoS**	Metal organic framework approach	Hollow structures of double-shelled CoS hollow nanoboxes	980<br>*118	1<br>1	---<br>39.9	---<br>756	---<br>10000	---<br>88	10
**$Co_{1-x}S$**	Hydrothermal	Flower like	674	3	---	---	1000	82.3	11
**$Co_{1-x}S$**	Hydrothermal	Hollow structure	420	1	19.4	2644	5000	---	12
**CoS**	Hydrothermal	Spheres	349	1	---		300	87	13
**CoS**	Microwave assisted heating	Hierarchical flower	586	1	20.4	6250	1000	91	14
**Co_9S_8**	Two-step hydrothermal route	Nano flakes<br>Octahedral	*83<br>*18.6	1.25<br>1	26.3-31.4<br>---	4000-200<br>---	5000<br>5000	89.5<br>65	15
**Co_3S_4**	Hydrothermal	Hierarchical porous nanocoral	1447<br>*132	1<br>1	---<br>60.1-38.5	---<br>418-3812	---<br>5000	---<br>90	16
**CoS**	Solvothermal	Dumb-bell shaped	310<br>*47	5<br>2	---<br>5.3	---<br>1800	5000<br>5000	95<br>92	4
**CoS**	**Hydrothermal**	**Hierarchical star anise structure**	**348**<br>***57**	**1**<br>**1**	**7.73-2.22**<br>**15.57-3.11**	**200-4000**<br>**700 -5600**	**1000**<br>**2000**	**97.2**<br>**97.9**	**Present work**

* Electrochemical parameters of two-electrode asymmetric supercapacitor device

References:

1 Prabhu, Y., Rao, K. , Kumar, V. and Kumari, B. X-Ray Analysis by Williamson-Hall and Size-Strain Plot Methods of ZnO Nanoparticles with Fuel Variation. *World Journal of Nano Science and Engineering* **4**, 21-28, doi:10.4236/wjnse.2014.41004 (2014).

2 Mote, V., Purushotham, Y. & Dole, B. Williamson-Hall analysis in estimation of lattice strain in nanometer-sized ZnO particles. *Journal of Theoretical and Applied Physics* **6**, 6, doi:10.1186/2251-7235-6-6 (2012).

3 Bindu, P. & Thomas, S. Estimation of lattice strain in ZnO nanoparticles: X-ray peak profile analysis. *Journal of Theoretical and Applied Physics* **8**, 123-134, doi:10.1007/s40094-014-0141-9 (2014).

4 Subramani, K., Sudhan, N., Divya, R. & Sathish, M. All-solid-state asymmetric supercapacitors based on cobalt hexacyanoferrate-derived CoS and activated carbon. *RSC Advances* **7**, 6648-6659, doi:10.1039/c6ra27331a (2017).

5 Wan, H. *et al.* Hydrothermal synthesis of cobalt sulfide nanotubes: The size control and its application in supercapacitors. *Journal of Power Sources* **243**, 396-402 (2013).

6 Subalakshmi, K. & Senthilselvan, J. Effect of fluorine-doped TiO_2 photoanode on electron transport, recombination dynamics and improved DSSC efficiency. *Solar Energy* **171**, 914-928, doi:https://doi.org/10.1016/j.solener.2018.06.077 (2018).

7 You, B., Jiang, N., Sheng, M. & Sun, Y. Microwave vs. solvothermal synthesis of hollow cobalt sulfide nanoprisms for electrocatalytic hydrogen evolution and supercapacitors. *Chemical Communications* **51**, 4252-4255, doi:10.1039/c4cc09849h (2015).

8 Xing, J.-C., Zhu, Y.-L., Zhou, Q.-W., Zheng, X.-D. & Jiao, Q.-J. Fabrication and shape evolution of CoS_2 octahedrons for application in supercapacitors. *Electrochimica Acta* **136**, 550-556, doi:https://doi.org/10.1016/j.electacta.2014.05.118 (2014).

9 Chen, H., Zhu, X., Chang, Y., Cai, J. & Zhao, R. 3D flower-like CoS hierarchitectures recycled from spent $LiCoO_2$ batteries and its application in electrochemical capacitor. *Materials Letters* **218**, 40-43, doi:https://doi.org/10.1016/j.matlet.2018.01.144 (2018).

10 Hu, H., Guan, Bu Y. & Lou, Xiong W. Construction of Complex CoS Hollow Structures with Enhanced Electrochemical Properties for Hybrid Supercapacitors. *Chem* **1**, 102-113, doi:https://doi.org/10.1016/j.chempr.2016.06.001 (2016).

11 Li, Y. *et al.* Facile synthesis of flower-like cobalt sulfide hierarchitectures with superior electrode performance for supercapacitors. *Journal of Alloys and Compounds* **712**, 139-146, doi:https://doi.org/10.1016/j.jallcom.2017.04.064 (2017).

12 Ranaweera, C. K. *et al.* Highly stable hollow bifunctional cobalt sulfides for flexible supercapacitors and hydrogen evolution. *Journal of Materials Chemistry A* **4**, 9014-9018, doi:10.1039/c6ta03158g (2016).

13 Justin, P. & Ranga Rao, G. CoS spheres for high-rate electrochemical capacitive energy storage application. *International Journal of Hydrogen Energy* **35**, 9709-9715, doi:https://doi.org/10.1016/j.ijhydene.2010.06.036 (2010).

14 Luo, F., Li, J., Yuan, H. & Xiao, D. Rapid synthesis of three-dimensional flower-like cobalt sulfide hierarchitectures by microwave assisted heating method for high-performance supercapacitors. *Electrochimica Acta* **123**, 183-189, doi:https://doi.org/10.1016/j.electacta.2014.01.009 (2014).

15 Rakhi, R. B., Alhebshi, N. A., Anjum, D. H. & Alshareef, H. N. Nanostructured cobalt sulfide-on-fiber with tunable morphology as electrodes for asymmetric hybrid supercapacitors. *Journal of Materials Chemistry A* **2**, 16190-16198, doi:10.1039/c4ta03341h (2014).

16 Liu, G. *et al.* Facile controlled synthesis of a hierarchical porous nanocoral-like Co_3S_4 electrode for high-performance supercapacitors. *RSC Advances* **6**, 54076-54086, doi:10.1039/c6ra10427d (2016).
